# The impact of the COVID-19 pandemic on melanoma diagnosis: a systematic review and meta-analysis of global evidence

**DOI:** 10.1186/s12889-025-23926-3

**Published:** 2025-08-06

**Authors:** Seyed Mostafa Mostafavi Zadeh, Elahe Noroozi, Elmira Gheytanchi, Fatemeh Tajik, Zahra Madjd, Davoud Ahmadvand

**Affiliations:** 1 Oncopathology Research Center, Iran University of Medical Sciences, Tehran, Iran; 2 Department of Molecular Medicine, Faculty of Advanced Technologies in Medicine, Iran University of Medical Sciences, Tehran, Iran; 3 Division of Gene Therapy, Gunma University Initiative for Advanced Research, Maebashi, Japan; 4 Department of Surgery, University of California, Irvine Medical Center, Orange, CA USA; 5 Department of Molecular Medicine, School of Advanced Technologies in Medicine, Iran University of Medical Sciences, Tehran, Iran; 6 Department of Molecular Imaging, Faculty of Advanced Technologies in Medicine, Iran University of Medical Sciences, Tehran, Iran

**Keywords:** Melanoma, COVID-19, Cancer diagnosis, Breslow thickness, Systematic review, Meta-analysis, Ulceration, Nodular melanoma

## Abstract

**Introduction:**

The COVID-19 pandemic significantly disrupted healthcare systems worldwide. Prioritizing emergency responses resulted in the postponement of routine medical care, including melanoma diagnoses. We performed a systematic review and meta-analysis to quantify the pandemic’s effect on diagnosis rates, Breslow thickness, stage at presentation, ulceration, histologic subtypes, and patient age.

**Method:**

We performed a systematic review and meta-analysis following PRISMA guidelines. PubMed, Scopus, Web of Science, and Embase were searched up to 10 September 2024 for observational studies comparing melanoma outcomes in the pre-COVID era (before March 2020) with the COVID era (March 2020 onwards). Two reviewers independently screened records, extracted data on diagnostic counts, patient age, Breslow thickness, ulceration, and histopathological subtype, and assessed study quality using the Newcastle–Ottawa Scale (NOS). Random-effects models pooled rate ratios (RRs) or odds ratios (ORs); fixed-effects models pooled mean differences (MDs). Heterogeneity was evaluated with I², and sensitivity analyses were restricted to high-quality studies (NOS ≥ 7).

**Results:**

Sixty-two studies (38,676 pre-COVID and 46,846 COVID-era melanomas) met inclusion criteria. New melanoma diagnoses fell by 19% during the pandemic (RR = 0.81, 95% CI 0.75–0.86; I² = 98%). Mean age at diagnosis rose by 0.86 years (95% CI 0.58–1.14; I² = 45%). Tumors were thicker (MD = 0.24 mm, 95% CI 0.02–0.47; I² = 92%) and more frequently ulcerated (OR = 1.29, 95% CI 1.15–1.44; I² = 31%). Nodular melanoma, an aggressive subtype, became more common (OR = 1.34, 95% CI 1.08–1.67; I² = 81%), whereas superficial spreading, acral lentiginous, and lentigo-maligna subtypes showed no significant change. All the key findings persisted in good-quality-only analyses.

**Conclusion:**

COVID-19-related service disruptions were associated with fewer melanoma diagnoses but a shift toward older patients and biologically adverse tumor features, signaling delayed detection at the population level. Strengthening resilient, rapid-access skin cancer pathways and integrating tele-dermatology with triaged in-person assessment are public-health priorities for future crises.

**Trial registration:**

PROSPERO registration number CRD42022361569.

**Supplementary Information:**

The online version contains supplementary material available at 10.1186/s12889-025-23926-3.

## Background

Towards the end of 2019, the outbreak of SARS-CoV-2, a highly contagious Coronavirus strain, marked the onset of a global health crisis. In response to its widespread impact, the World Health Organization (WHO) declared a pandemic in March 2020 [1]. COVID-19, caused by SARS-CoV-2, is primarily transmitted through respiratory droplets and close interactions with infected individuals [2]. The pandemic claimed millions of lives worldwide and significantly impacted countries across the globe. Various measures were implemented to control the spread of the virus, including isolation and quarantine of infected individuals, as well as restrictions on social activities [3, 4]. These measures profoundly affected the global economy, mental health, and healthcare systems [5].

Healthcare systems worldwide prioritized COVID-19-related care, focusing on emergency supplies and critical cases. Consequently, non-urgent services including routine cancer screening and follow-up were deferred or cancelled [6]. Routine screenings and non-urgent appointments were often postponed or canceled as healthcare professionals across specialties redirected their efforts to manage COVID-19 patients. Furthermore, fear of contracting COVID-19 discouraged individuals from attending routine screenings, exacerbating delays in cancer diagnosis [7, 8]. For instance, breast- and colorectal-cancer screening volumes in 20 U.S. centers fell by 89% and 85% in April 2020 versus April 2019 [6]. Similarly, a study in Italy found that cancer diagnosis rates dropped by 45% during weeks 11 to 20 of 2020 compared to the same period in 2019 [9].

Although non-melanoma skin cancers are numerically dominant, melanoma accounts for the majority of skin-cancer deaths because of its aggressive biology. GLOBOCAN 2022 lists melanoma 17th worldwide, with almost 332,000 new cases and 58,700 deaths annually [10, 11]. The global burden of melanoma is estimated to reach 510,000 new cases (an increase of roughly 50%) and 96,000 deaths (an increase of 68%) by 2040 if current trends persist [12].

The prognosis for melanoma varies markedly based on the stage at diagnosis. The five-year survival rate in the U.S. exceeds 99% when melanoma is detected early, but drops to 74% when the disease spreads to the lymph nodes, and plummets to 35% upon metastasis to distant organs [11]. The remarkable difference between these survival rates highlights that early detection and timely management of melanoma considerably improve patient outcomes.

Early detection of melanoma is critical for reducing disease burden, as prognosis strongly depends on tumor thickness and stage at diagnosis [13, 14]. Screening programs aim to facilitate earlier diagnosis; however, most have demonstrated benefits in terms of identifying thinner melanomas rather than directly reducing mortality. The SCREEN project in Northern Germany is the only large-scale population-based initiative to date that has shown a significant reduction in melanoma mortality following systematic screening [15]. Visual inspection and dermoscopy remain essential tools for detecting suspicious lesions at an early stage, thereby improving the likelihood of favorable outcomes [16, 17]. Early diagnosis of melanoma is critical, since delayed diagnosis is strongly associated with increased Breslow thickness, which has been shown to be an important histopathological indicator of survival. Melanoma-specific death, metastasis, and nodal involvement are more likely to occur in thicker tumors. Several studies have shown that physician-detected melanomas are diagnosed at significantly lower Breslow thickness compared to patient-detected lesions. This likely reflects earlier-stage detection enabled by routine full-body exams and dermoscopy [13, 14, 18].

During the initial months of the COVID-19 pandemic in 2020, the rate of melanoma diagnoses in the U.K. and the U.S. declined significantly [19, 20]. A global survey conducted by the Global Coalition for Melanoma Patient Advocacy among dermatologists from 36 countries estimated that 21% of melanoma cases went undiagnosed during the pandemic [21]. These findings indicate that delays in follow-up care likely contributed to an increase in advanced melanoma cases and poorer prognosis. Reduced access to medical care and necessary equipment, as well as concerns about COVID-19 transmission among patients, may have further discouraged dermatologist visits. To address these challenges, healthcare systems adopted innovative approaches, including telemedicine, to maintain patient care. Despite these efforts, the pandemic caused substantial delays in melanoma diagnosis, with implications for disease outcomes [22]. Disruptions in healthcare delivery during the COVID-19 pandemic including reduced access to in-person dermatologic care, diagnostic delays, and variable adoption of teledermatology, may have significantly influenced melanoma detection patterns and outcomes [20, 23]. 

The aim of this systematic review and meta-analysis is to assess how the COVID-19 pandemic affected melanoma diagnosis by comparing pre-COVID and COVID-era data. Specifically, we examine changes in diagnostic volume, Breslow thickness, ulceration, clinical stage at presentation, histopathological subtypes, and patient age to understand how care disruptions may have influenced disease severity and presentation.

## Methods

### Protocol registration

This systematic review adheres to the Preferred Reporting Items for Systematic Reviews and Meta-Analyses (PRISMA) Checklist guidelines [24]. The study protocol has been registered in the PROSPERO database (registration number: CRD42022361569) and published [25].

### Study design and selection criteria

This study aimed to evaluate the impact of the COVID-19 pandemic on melanoma diagnosis by comparing data from the pandemic period (2020 onward) with the pre-pandemic era. The research question was structured using the PECO framework as follows:

- **Population (P)**: Individuals of all ages and genders diagnosed with cutaneous melanoma during the specified time periods.
- **Exposure (E)**: The COVID-19 pandemic period (2020 onward).
- **Comparator (C)**: The pre-COVID era (prior to 2020).
- **Outcome (O)**: Changes in melanoma diagnosis rates, stage at diagnosis, Breslow thickness, ulceration, and histopathological subtypes.

**Inclusion Criteria**:

Studies were included if they:

- Investigated individuals of all ages and genders diagnosed with cutaneous melanoma.
- Used observational study designs such as cross-sectional, cohort, or case-control studies, as well as letters. While letters are not typically featured in systematic reviews due to limited methodological detail and less rigorous peer review, their inclusion was warranted by the exceptional circumstances surrounding the COVID-19 pandemic, which led to a surge of findings being published in this format. To ensure methodological transparency, all included letters underwent quality assessment using the Newcastle–Ottawa Scale (NOS).
- Reported outcomes related to melanoma diagnosis, such as stage at diagnosis, number of diagnoses, histopathological subtypes, laboratory or pathological findings, participation rates, and diagnostic assessments during the pre-COVID-19 and COVID-19 periods.

**Exclusion Criteria**:

Studies were excluded if they:

- Focused on non-melanoma skin cancers or other malignancies.
- Involved in vivo or in vitro experimental research.
- Were reviews, meta-analyses, commentaries, errata, corrigenda, case reports, case series, editorials, or book chapters.
- Had insufficient data or lacked access to the full text.
- Were published in languages other than English.

### Search strategy

A thorough literature search was conducted to identify studies on melanoma diagnosis during the COVID-19 era. Searches were performed in four primary databases- PubMed/MEDLINE, Scopus, Web of Science (WOS), and Embase on September 10, 2024. The searches utilized a combination of keywords and Medical Subject Headings (MeSH) terms such as “Melanoma”, “Skin Neoplasms” [Mesh Terms], “Malignant melanoma”, “skin cancer”, “COVID-19” [Mesh Terms], and “SARS-CoV-2” was used, along with Boolean operators (AND, OR) to combine terms. Modifications were made to adapt the search syntax for different databases. Additionally, manual searches of reference lists from included studies were conducted to identify further relevant articles. Only studies published in English were included. No restrictions were applied regarding publication date or study setting. The search strategy is available in the Supplementary Table S1.

### Selection process

After the initial search, all identified records were exported to EndNote software (version 21.2, Clarivate, Philadelphia, USA). The selection process involved three steps. First, duplicate entries were removed using EndNote and through manual review. In the second step, the remaining studies were then screened by two independent reviewers (SMMZ and EN) based on predefined criteria for eligibility. Finally, two authors (EGh and EN) independently conducted a full-text review of the selected studies. A third reviewer (SMMZ) was consulted, when necessary, in order to resolve any discrepancies between the reviewers.

### Data extraction

Two reviewers (EN and DA) independently extracted data using a pre-piloted Excel form. The tabulated dataset includes bibliographic information (first author, publication year), country, era classification (pre-COVID and COVID), exact period definitions, and the duration (in months) of each era. For each era, the number of newly diagnosed melanoma cases was recorded, along with male-to-female ratios when reported. Study quality was assessed using the Newcastle–Ottawa Scale (NOS), and scores were converted into AHRQ categories (Good, Fair, Poor). This structured data is summarized in Supplementary Table S2.

In addition to the data presented in the Supplementary Table S2, clinical variables used for meta-analyses were extracted separately, including mean age (± SD), Breslow thickness (mean ± SD), melanoma stage at diagnosis, ulceration status, and histopathological subtypes.

### Quality assessment

Two authors (EN and FT) evaluated the methodological quality of the included studies utilizing the Newcastle-Ottawa Scale (NOS) checklist. The checklist comprises three sections: participant selection, group comparability, and outcome assessment [26]. The criteria assign a maximum of four stars for the selection domain, two stars for the comparability domain, and three stars for the exposure (in case-control studies) or outcomes (in cohort studies) domain. Study quality was classified based on the Agency for Healthcare Research and Quality (AHRQ) standards, using the distribution of stars across the three domains:

- Good quality required 3 or 4 stars in the selection domain, 1 or 2 stars in the comparability domain, and 2 or 3 stars in the outcome/exposure domain;
- Fair quality required 2 stars in the selection domain, 1 or 2 stars in the comparability domain, and 2 or 3 stars in the outcome/exposure domain;
- Poor quality was assigned to studies with 0 or 1 star in the selection domain, or 0 stars in the comparability domain, or 0 or 1 star in the outcome/exposure domain [27].

Any disagreements between the two authors were resolved through consensus and subsequently reviewed by a third author (ZM).

### Data synthesis

Although initial heterogeneity in study designs, populations, and diagnostic approaches was anticipated, sufficient comparable data enabled meta-analyses using random-effects models (Mantel-Haenszel for odds ratios, fixed-effects for mean differences) for key outcomes. Meta-analyses were conducted for diagnosis rates (62 datasets), mean age (16 datasets), Breslow thickness (13 datasets), ulceration (20 datasets), and histopathological subtypes (varying datasets), with heterogeneity assessed using Tau², Chi² test, and I² statistics. To address variability in definitions of pre-COVID and COVID periods across studies, ranging from 2011 to early 2023, we standardized the categorization for consistency. The COVID era was defined as any period starting in March 2020 or later, in alignment with the World Health Organization’s pandemic declaration on March 11, 2020 [1], and the ensuing implementation of global containment measures. Periods ending before March 2020 were designated as the pre-COVID era. This cutoff was selected because most included studies adopted March 2020 as the onset of pandemic-related disruptions to healthcare, aligning with the start of significant changes in melanoma diagnostic practices. Pooled estimates (odds ratios or mean differences) were presented with corresponding 95% confidence intervals and Z-statistics, with statistical significance determined using P-values (threshold *P* < 0.05). Findings were visualized in forest plots for each outcome. A single summary table was constructed to present study-level details, including first author, year, country, sample size, gender distribution, study quality (NOS), and period definitions (Supplementary Table 2). Sensitivity analyses were conducted for each outcome by excluding poor-quality and fair-quality studies and, where applicable, restricting analyses to those rated as “Good” quality to evaluate the robustness of findings and assess the influence of study quality on pooled estimates.

## Results

Following our comprehensive data synthesis approach outlined in the Methods section, we identified significant changes in melanoma diagnosis patterns during the COVID-19 era, reflecting the impact of healthcare disruptions. Below, we present the findings, starting with the reduction in new melanoma diagnoses, followed by changes in patient demographics and tumor characteristics.

### Study selection

A comprehensive database search was initially conducted on March 30, 2024, and then updated on September 10, 2024, yielding a total of 5671 records from PubMed (*n* = 687), Scopus (*n* = 2392), Web of Science (*n* = 835), and Embase (*n* = 1757). Following the removal of 2133 duplicates using EndNote and an additional 239 duplicates identified manually, 3299 unique studies proceeded to the primary screening phase.

Screening these records based on title and abstract resulted in the exclusion of 1550 records deemed irrelevant to the research question, 1403 that did not match the required study type, and one retracted publication. Consequently, 345 records underwent full-text retrieval. Of these, 25 articles could not be obtained, leaving 320 that were assessed in detail.

**Fig. 1:**
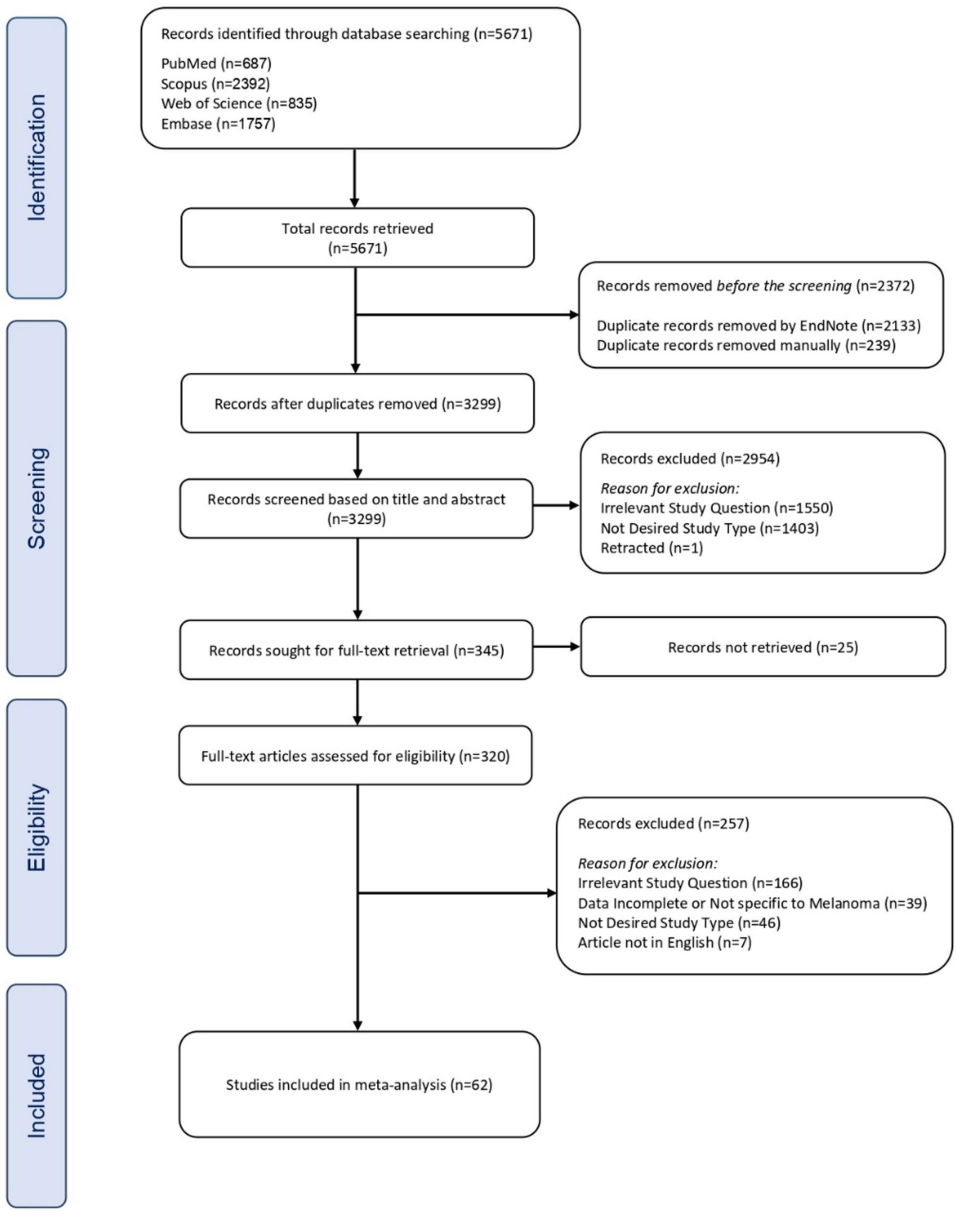
Flowchart for the search strategy according to the PRISMA guidelines

During the full-text review, 166 articles were excluded as irrelevant to the study question, 39 for incomplete or non–melanoma–specific data, 46 did not meet the inclusion criteria for study design, and seven were in languages other than English. Following these exclusions, 62 studies met all inclusion criteria and were included in the quantitative meta-analysis. All included papers employed a retrospective cohort design. The study selection process was reported by PRISMA (Preferred Reporting Items for Systematic Reviews and Meta-Analyses) guidelines as illustrated in the flow diagram presented in Fig. 1.

### Study characteristics

The meta-analysis included 62 studies, identified through a systematic search of databases including PubMed (*n* = 687), Scopus (*n* = 2392), Web of Science (*n* = 835), and Embase (*n* = 1757), yielding a total of 5671 records. After removing 2372 duplicates (2133 via EndNote and 239 manually), 3299 records remained. Following screening, 2954 records were excluded for reasons such as irrelevant study questions (*n* = 1550), undesired study types (*n* = 1403), or retraction (*n* = 1). Of the 345 records sought for full-text retrieval, 25 could not be retrieved, leaving 320 full-text articles for eligibility assessment. A further 257 were excluded due to irrelevant study questions (*n* = 166), incomplete or non-specific melanoma data (*n* = 39), undesired study types (*n* = 46), or non-English articles (*n* = 7), resulting in 62 studies included in the meta-analysis.

These studies collectively reported on 33,846 melanoma diagnoses in the COVID era and 38,676 in the pre-COVID era, spanning multiple countries including Romania, Canada, Germany, Brazil, Italy, Ireland, Belgium, the USA, Turkey, the UK, Spain, France, Austria, Switzerland, Serbia, Portugal, the Netherlands, Scotland, North Cancer Alliance, Chile, Greece, and Poland. The studies varied significantly in their definitions of the pre-COVID and COVID eras, with some extending into a post-COVID period.

The data collection periods across the included studies range from 1 January 2015 to 28 February 2023, as detailed in Supplementary Table S2. To standardize the categorization for meta-analysis, the COVID era was defined as any period beginning on or after March 2020, while the pre-COVID era included periods ending before March 2020, as mentioned earlier. The duration of study periods ranged from 1 to 38.5 months, with sample sizes varying from 3 to 63,087 newly diagnosed melanoma cases. Gender distribution was reported in 21 studies, with male-to-female ratios ranging from 0.80 to 1.66.

### Quality assessment of studies

The quality of the included studies was assessed using the Newcastle-Ottawa Scale (NOS), which evaluates cohort studies based on selection, comparability, and outcome. Scores ranged from 4 to 9, with 37 studies classified as good quality (NOS ≥ 7), 12 as fair (NOS 6), and 14 as poor (NOS ≤ 5). To align with reporting standards, the NOS scores were converted to the Agency for Healthcare Research and Quality (AHRQ) standards, categorizing studies as good (NOS 7–9), fair (NOS 6), or poor (NOS ≤ 5). This conversion ensured consistency in quality reporting across studies. We performed sensitivity analyses based on NOS-derived quality ratings, excluding Poor-quality studies and separately restricting to Good-quality studies, to confirm that our pooled estimates remained robust to study quality.

### Change in the number of newly diagnosed melanomas

**Fig. 2:**
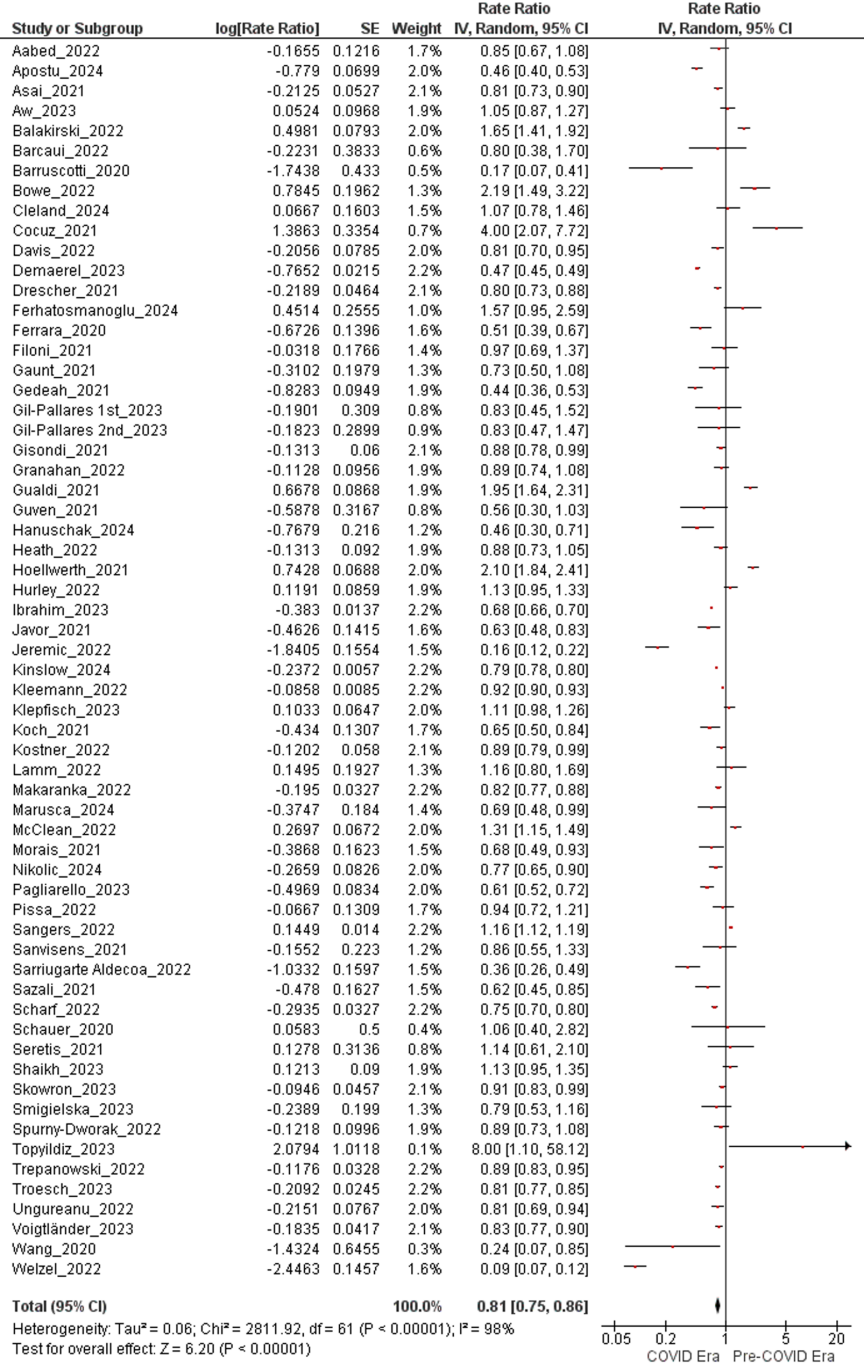
Forest plots showing the rate ratio (RR) of newly diagnosed melanomas during the COVID era compared to the pre-COVID era. Analyses were conducted using a random-effects model. Horizontal lines indicate 95% confidence intervals

The meta-analysis revealed a statistically significant reduction in new melanoma diagnoses during the COVID era compared to the pre-COVID era. A total of 62 studies contributed 61 independent datasets (Gil-Pallares 2023 provided two non-overlapping six-month intervals and was therefore entered twice). Altogether, these studies reported 38,676 incident melanomas in the pre-COVID era and 33,846 in the COVID era. Random-effects synthesis yielded a rate ratio (RR) of 0.81 (95% CI 0.75–0.86; Z = 6.20; *P* < 0.00001), indicating a 19% decline in new melanoma diagnoses after March 2020 (Fig. 2). Statistical heterogeneity was considerable (τ² = 0.06; χ² = 2,811.92, df = 61; *P* < 0.00001; I² = 98%), consistent with differences in geography, data sources, and period definitions across studies.

To appraise the influence of study quality, a prespecified sensitivity analysis was performed that was limited to the 29 studies graded “Good” on the Newcastle–Ottawa Scale. The resulting estimate remained directionally consistent—RR = 0.88 (95% CI 0.80–0.97; Z = 2.65; *P* = 0.008)—indicating that the observed decline persists even when the synthesis is restricted to the most methodologically robust evidence.

#### Geographical heterogeneity

**Fig. 3:**
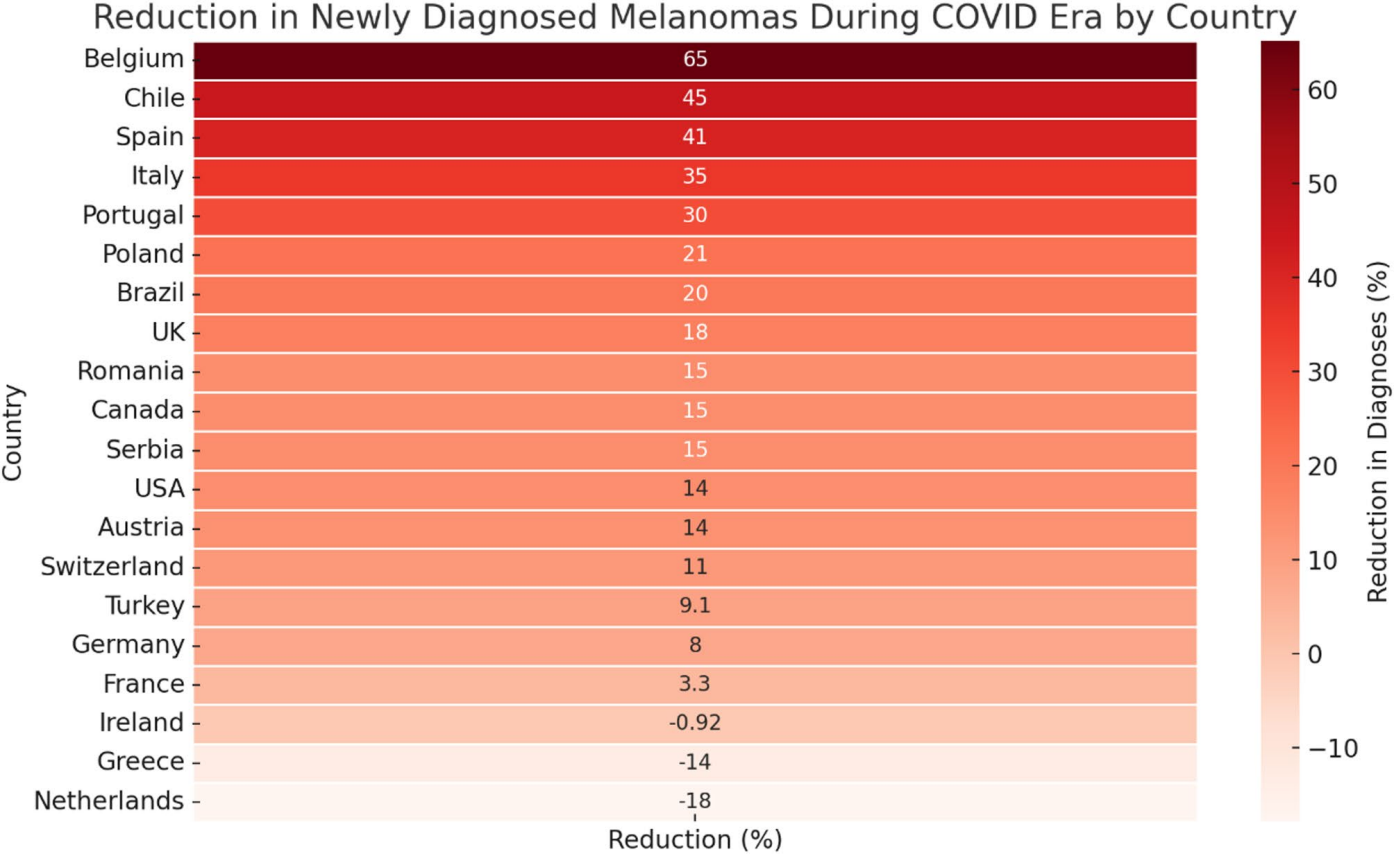
Percentage change in newly diagnosed melanomas during the COVID-19 era versus the pre-COVID era, stratified by country. Positive values denote reductions; negative values represent net increases across the study window

The absolute reduction in newly diagnosed melanomas during the COVID-19 era varied strikingly between national datasets (Fig. 3). Among the 20 countries represented, the median decline was 15% (inter-quartile range 14–23%), with point estimates spanning from a 65% shortfall to an 18% net increase. The steepest decreases were documented in Belgium (65%) [28, 29], Chile (45%) [30], Spain (41%) [31–34], and Italy (35%) [9, 35–39]. Portugal, Poland, Brazil, and the United Kingdom experienced intermediate reductions of 15–30% [40–43], whereas France registered only a marginal decline (3%) [44–46]. By contrast, three countries recorded net rebounds, with Ireland (~ 1% increase) [47–49], Greece (14% increase) [50], and the Netherlands (18% increase) [22] surpassing their pre-pandemic diagnostic volumes, a pattern compatible with catch-up activity once services resumed. This pronounced geographical dispersion accounted for much of the residual heterogeneity in the pooled analysis (I² = 98%).

### Mean age

**Fig. 4:**
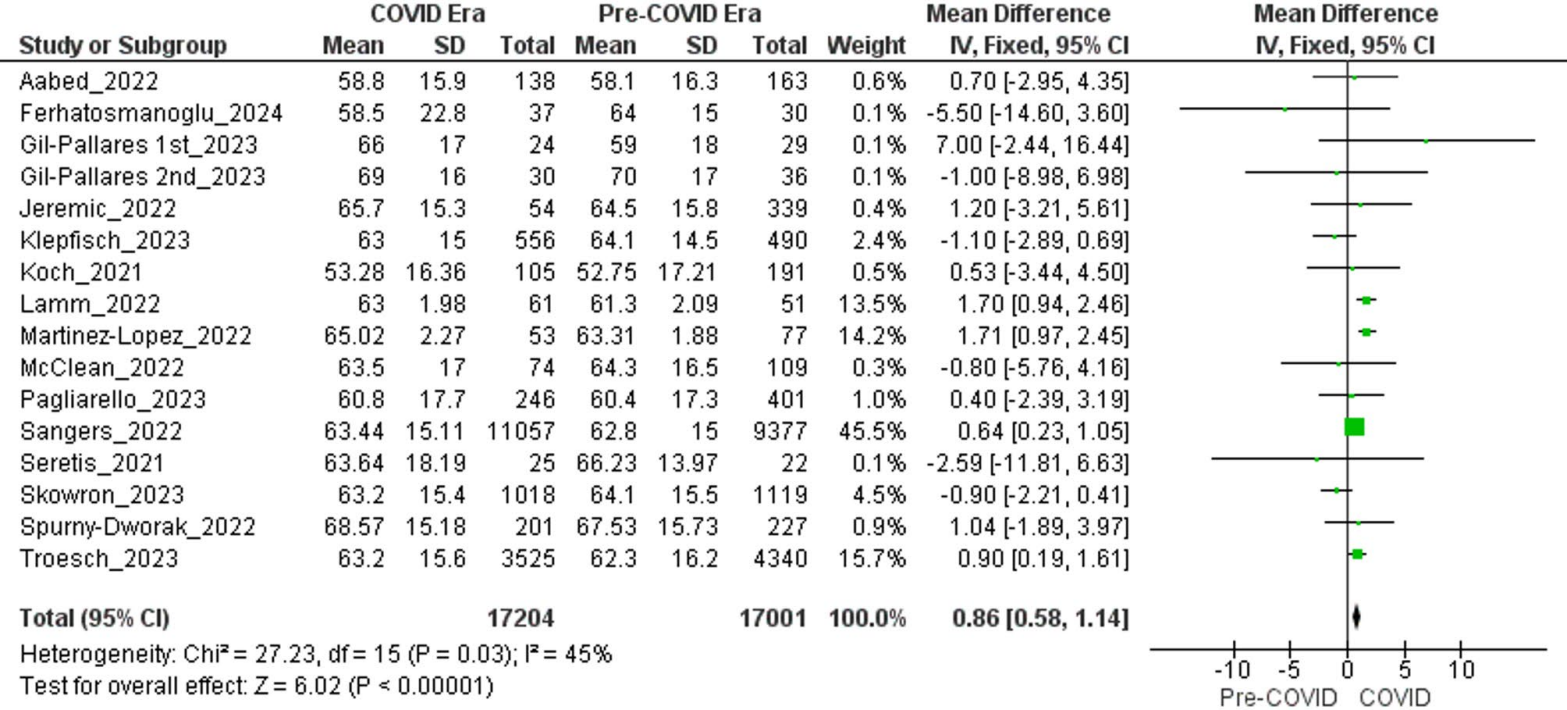
Forest plots comparing the mean age at melanoma diagnosis during the COVID era versus the pre-COVID era

The meta-analysis detected a small but statistically significant rise in the mean age at melanoma diagnosis during the pandemic. Fifteen primary studies contributed 16 independent datasets (Gil-Pallares, 2023 [31] incorporated two non-overlapping six-month intervals that were analyzed separately). In aggregate, these datasets comprised 17,001 patients in the pre-COVID era and 17,204 in the COVID era. Fixed-effects pooling produced a mean difference (MD) of 0.86 years (95% CI 0.58–1.14; Z = 6.02; *P* < 0.00001), indicating that melanomas diagnosed after March 2020 occurred, on average, in slightly older individuals (Fig. 4). Between-study heterogeneity was moderate (χ² = 27.23, df = 15; *P* = 0.03; I² = 45%), reflecting variation in sampling frames and healthcare settings. Individual study effects ranged from − 2.59 years (95% CI − 4.35 to − 0.95) to + 2.65 years (95% CI 1.88 to 3.41).

To examine the influence of study quality, a prespecified sensitivity analysis was restricted to the 10 datasets derived from investigations rated “Good” on the Newcastle–Ottawa Scale. The association not only persisted but was slightly larger—MD = 0.99 years (95% CI 0.70–1.29; Z = 6.86; *P* < 0.00001)—and residual heterogeneity diminished (χ² = 13.03, df = 9; *P* = 0.16; I² = 31%). Hence, the upward shift in age at diagnosis is robust and cannot be attributed to low-quality evidence.

### Breslow thickness

The meta-analysis revealed a modest but statistically significant increase in Breslow thickness—a key indicator of melanoma severity—during the COVID era compared with the pre-COVID era. We initially included 14 datasets (from 13 unique studies—Gil-Pallares 2023 contributed two non-overlapping six-month intervals). One dataset (Seretis_2021) [50] was an extreme outlier (MD − 5.57 mm; 95% CI − 7.46 to − 3.68) and thus excluded.

The primary analysis then comprised 13 datasets (12 unique studies) and yielded a pooled mean difference of 0.24 mm (95% CI 0.02–0.47; Z = 2.10; *P* = 0.04). Substantial heterogeneity was present (Tau² = 0.11; Chi² = 158.46, df = 12, *P* < 0.00001; I² = 92%), likely reflecting variation in populations and pandemic-related diagnostic delays. Individual study MDs ranged from − 0.60 mm (95% CI − 1.45 to 0.25) to 1.18 mm (95% CI 0.27 to 2.09).

**Fig. 5:**
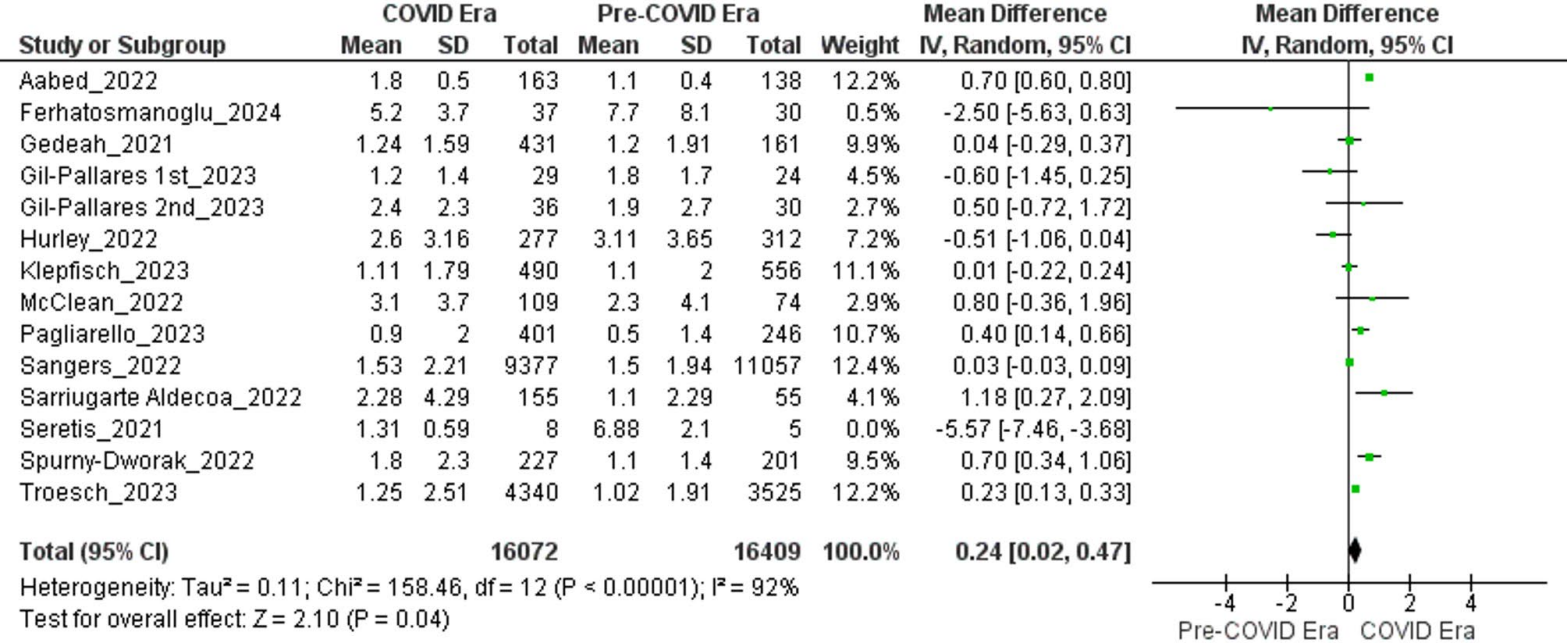
Forest plots comparing Breslow thickness of melanomas diagnosed during the COVID era versus the pre-COVID era (excluding Seretis as an outlier)

Restricting the pool to the seven studies rated “Good” on the Newcastle–Ottawa Scale yielded a comparable estimate—MD = 0.22 mm (95% CI 0.04–0.40; *Z* = 2.40; *P* = 0.02) with reduced, though still high, heterogeneity (*I*² = 85%). However, Seretis 2021 persisted as a pronounced outlier and therefore its removal left six high-quality datasets (14 427 COVID-era and 15 070 pre-COVID melanomas) and modestly amplified the effect (MD = 0.34 mm; 95% CI 0.01–0.66; Z = 2.05; *P* = 0.04), while heterogeneity remained elevated (τ² = 0.11; χ² = 132.77, df = 5; *P* < 0.00001; I² = 96%) (Fig. 5).

Thus, even under the strictest limitations to good-quality studies and excluding implausible outliers, COVID-era melanomas were, on average, approximately 0.3 mm thicker than those diagnosed beforehand. The high residual heterogeneity (*I*² ≈ 96%) indicates that effect size varied widely among settings (likely reflecting differences in healthcare disruption and case-mix) but the direction of the association is robust.

### Ulceration

**Fig. 6:**
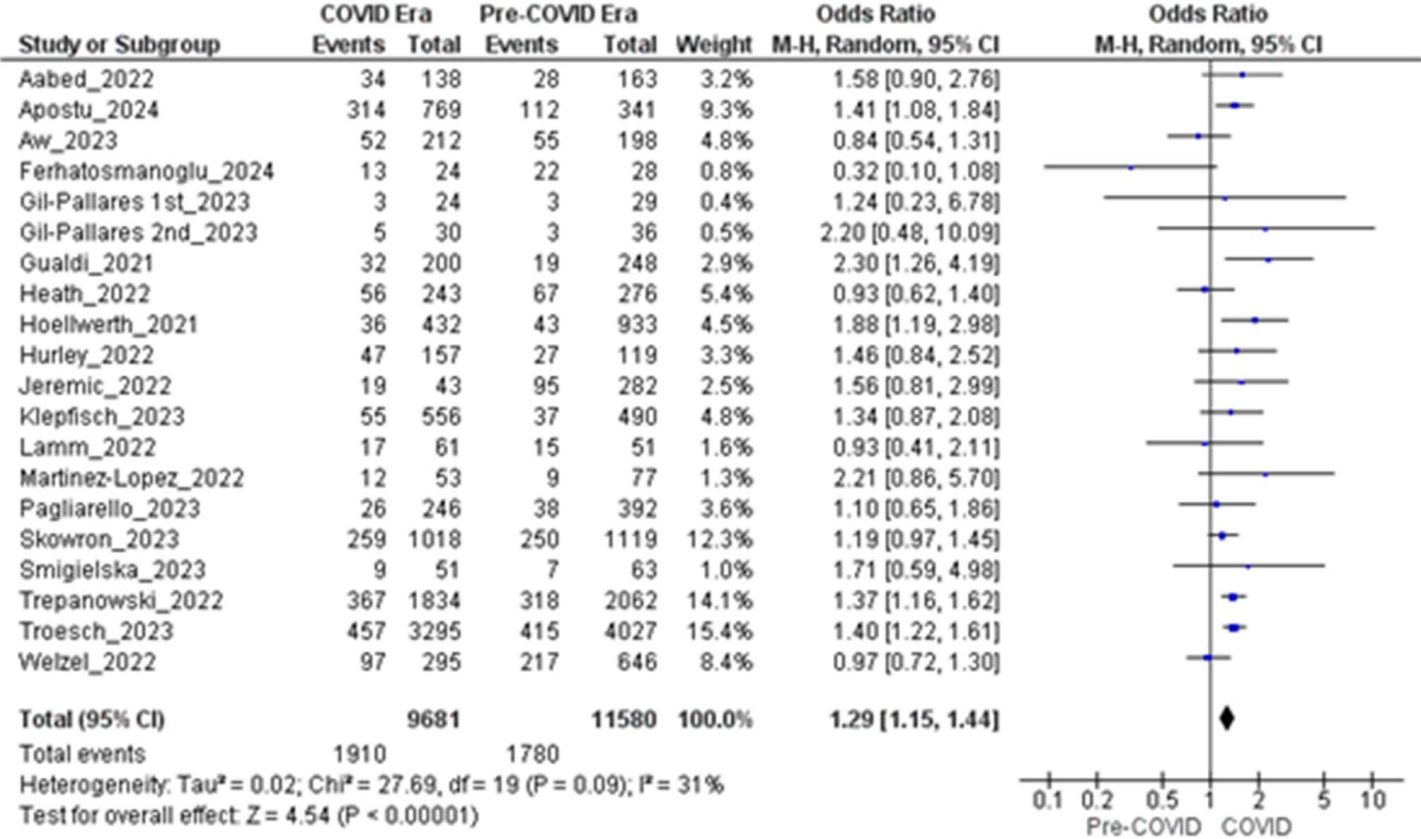
Forest plot comparing the proportion of ulcerated melanomas diagnosed in the COVID-19 vs. pre-COVID era

Twenty independent datasets from nineteen cohorts (Gil-Pallares 2023 provided two non-overlapping six-month intervals) met the inclusion criteria, contributing 21,261 melanomas to the analysis. During the COVID-19 period, 1,910 of 9,681 tumors (19.7%) were ulcerated, compared to 1,780 of 11,580 tumors (15.4%) in the pre-COVID period. A Mantel–Haenszel random-effects model revealed a significant association, with a pooled odds ratio (OR) of 1.29 (95% CI: 1.15–1.44; Z = 4.54; *P* < 0.00001). Between-study heterogeneity was low to moderate (τ² = 0.02; χ² = 27.69, df = 19, *P* = 0.09, I² = 31%), with individual study ORs ranging from 0.84 to 2.30 (Fig. 6).

A sensitivity analysis, restricted to twelve datasets rated “Good” on the Newcastle–Ottawa Scale (7,307 COVID-era and 8,802 pre-COVID melanomas; 1,379 vs. 1,169 ulcerated), confirmed and slightly strengthened the effect (OR = 1.37, 95% CI: 1.18–1.58; Z = 4.25; *P* < 0.00001). Heterogeneity remained comparable (τ² = 0.02; χ² = 17.81, df = 11, *P* = 0.09, I² = 38%). Influence diagnostics showed that excluding any single dataset altered the pooled OR by less than 0.05, without impacting statistical significance. These findings suggest that melanomas diagnosed during the pandemic were approximately one-third more likely to present with ulceration, potentially indicating more advanced disease. This shift may be linked to diagnostic delays and limited access to routine skin examinations during COVID-19-related healthcare disruptions.

### Histopathological subtypes

Among all histopathological subtypes, nodular melanoma showed a statistically significant increase during the COVID era. The primary analysis, which included 13 studies, identified 1,466 events in the COVID era and 1,395 in the pre-COVID era, yielding an odds ratio (OR) of 1.34 (95% CI: 1.08–1.67). This association was statistically significant (Z = 2.64, *P* = 0.008), and heterogeneity was modest (Tau² = 0.11; Chi² = 63.19, df = 12, *P* < 0.00001; I² = 81%), indicating some variability in effect estimates across studies. A sensitivity analysis was conducted on 10 studies rated good-quality using the Newcastle–Ottawa. This subset reported 1,268 events in the COVID era and 1,190 in the pre-COVID era, yielding an elevated odds ratio of 1.47 (95% CI: 1.13–1.91). The result remained statistically significant (Z = 2.85, *P* = 0.004), although heterogeneity increased (Tau² = 0.13; Chi² = 57.77, df = 9, *P* < 0.00001; I² = 84%), likely due to differences in healthcare accessibility, referral patterns, and regional pandemic impact.

In contrast, superficial spreading melanoma showed no statistically significant change during the COVID era. The primary analysis, which included 13 studies, identified 4,335 events in the COVID era and 4,723 in the pre-COVID era, yielding an odds ratio of 0.94 (95% CI: 0.84–1.07). This association was not statistically significant (Z = 0.91, *P* = 0.36), and heterogeneity was moderate (Tau² = 0.02; Chi² = 26.34, df = 12, *P* = 0.010; I² = 54%). A sensitivity analysis was conducted on 10 studies rated good-quality using the Newcastle–Ottawa. This subset reported 3,906 events in the COVID era and 4,723 in the pre-COVID era, yielding an odds ratio of 0.91 (95% CI: 0.79–1.04). The result remained statistically non-significant (Z = 1.35, *P* = 0.18), with similar heterogeneity (Tau² = 0.02; Chi² = 16.86, df = 8, *P* = 0.03; I² = 53%).

Likewise, acral lentiginous melanoma showed no statistically significant change. The primary analysis, which included 6 studies, identified 139 events in the COVID era and 133 in the pre-COVID era, yielding an odds ratio of 1.13 (95% CI: 0.89–1.44). This association was not statistically significant (Z = 1.00, *P* = 0.32), and heterogeneity was absent (Tau² = 0.00; Chi² = 1.31, df = 5, *P* = 0.93; I² = 0%). A sensitivity analysis was conducted on 4 studies rated good-quality using the Newcastle–Ottawa. This subset reported 99 events in both the COVID and pre-COVID eras, yielding an odds ratio of 1.08 (95% CI: 0.81–1.44). The result remained non-significant (Z = 0.52, *P* = 0.61), with unchanged heterogeneity (Tau² = 0.00; Chi² = 0.75, df = 3, *P* = 0.86; I² = 0%).

**Fig. 7:**
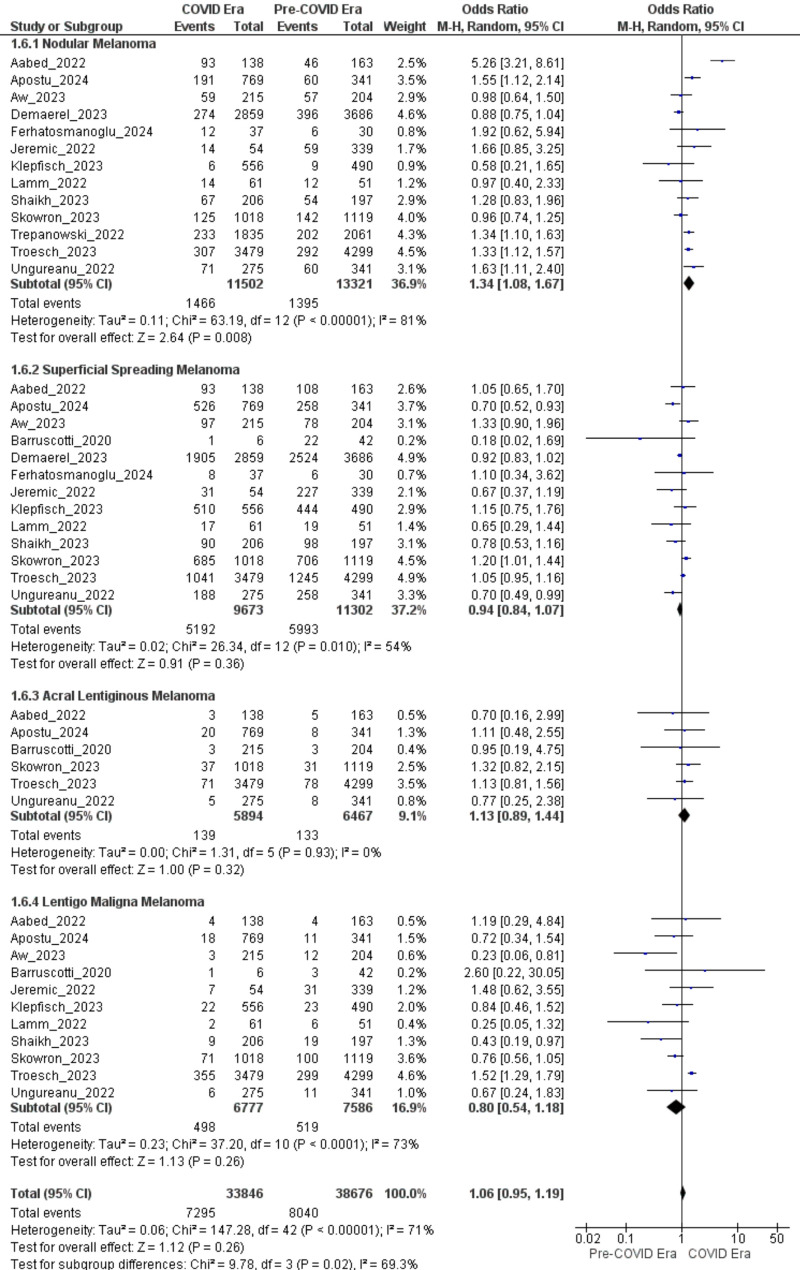
Forest plots comparing the odds of histopathological melanoma subtypes diagnosed during the COVID era versus the pre-COVID era

Lastly, lentigo maligna melanoma showed no statistically significant change during the COVID era. The primary analysis, which included 11 studies, identified 498 events in the COVID era and 519 in the pre-COVID era, yielding an odds ratio of 0.80 (95% CI: 0.54–1.18). This association was not statistically significant (Z = 1.13, *P* = 0.26), and heterogeneity was substantial (Tau² = 0.23; Chi² = 37.20, df = 10, *P* < 0.0001; I² = 73%). A sensitivity analysis was conducted on 7 studies rated good-quality using the Newcastle–Ottawa. This subset reported 395 events in the COVID era and 374 in the pre-COVID era, yielding an odds ratio of 0.82 (95% CI: 0.47–1.43). The result remained statistically non-significant (Z = 0.70, *P* = 0.49), and heterogeneity remained high (Tau² = 0.31; Chi² = 18.00, df = 6, *P* = 0.006; I² = 67%). (Fig. 7)

## Discussion

The COVID-19 pandemic profoundly disrupted global healthcare systems, redirecting resources toward emergency responses and significantly impacting routine cancer care, including melanoma diagnosis. This systematic review and meta-analysis, encompassing 62 studies and over 72,000 melanoma cases, provides robust evidence of the pandemic’s detrimental effects on melanoma diagnostic practices. Our findings reveal a 19% reduction in new melanoma diagnoses, a modest increase in mean age at diagnosis, thicker tumors (increased Breslow thickness), higher ulceration rates, and a shift toward more aggressive histopathological subtypes, particularly nodular melanoma. These changes collectively suggest a concerning trend toward delayed diagnoses and more advanced disease presentations. This has potential implications for long-term patient outcomes and healthcare system resilience.

### Geographical heterogeneity and system-level determinants

The heat map (Fig. 3) demonstrates significant variation in melanoma diagnosis rates across countries, with Belgium experiencing a 65% decrease and the Netherlands [51] seeing an 18% overall increase. This pattern can be explained by a combination of pandemic severity, changes in healthcare services, and digital preparedness. Belgium, Spain, and Italy, which were among the first and hardest-hit nations, enforced extended and stringent lockdowns, leading to the most significant drop in melanoma diagnoses. This aligns with registry-based findings that associate the peak lockdown period in April 2020 with a 40–45% reduction in newly diagnosed invasive cancer diagnoses [52, 53]. 

In contrast, nations with well-established teledermatology infrastructure (e.g., the Netherlands) [54] or rapid re-establishment of elective services (France, Ireland) [49, 55] displayed negligible declines or even compensatory increases, suggesting that digital triage and early service restoration can reduce diagnostic disruption.

Intermediate reductions in Poland, Brazil, and the UK probably reflect heterogeneous regional restrictions and phased re-openings. This large variation between countries likely contributes to the high statistical heterogeneity (I² ≈ 98%) in our pooled analysis, highlighting the risk of applying overall estimates to specific health systems.

### Reduction in melanoma diagnoses

The 19% drop in melanoma diagnoses observed during the COVID-19 period (RR = 0.81, 95% CI: 0.75–0.86) corresponds with global reports of decreased cancer screening and diagnostic activities during the pandemic [6, 9]. This decline is likely due to several factors: healthcare systems focusing on COVID-19 care, suspending routine dermatological screenings, and patients’ unwillingness to seek medical help because of fear of infection or lockdown measures. Sensitivity analyses limited to studies deemed high-quality (RR = 0.88, 95% CI: 0.80–0.97) confirmed the reliability of this finding, although significant heterogeneity (I² = 98%) indicates differences in regional healthcare disruptions, lockdown severity, and study methodologies. The reduction in diagnoses was especially notable in areas with strict lockdowns, such as Italy and Spain, where studies reported decreases of up to 45% [9]. This likely led to the underdiagnosis of early-stage melanomas, as shown by the reported decline in melanoma in situ (MIS) compared to invasive melanoma in several studies [32].

### Shift in patient demographics

A small but significant increase in the average age at diagnosis (+ 0.86 years, 95% CI: 0.58–1.14) suggests that younger patients, who frequently discover their conditions during routine or preventative check-ups, were disproportionately impacted by limited access to healthcare. This observation was consistent across good-quality studies (+ 0.99 years, 95% CI: 0.70–1.29), with moderate heterogeneity (I² = 45%) suggesting some differences in patient demographics and healthcare environments. Older patients, who may have more pronounced symptoms or advanced conditions, were likely more inclined to seek medical attention despite the obstacles posed by the pandemic. In contrast, younger, asymptomatic individuals postponed their visits. This shift in age could affect treatment choices, as older patients often have additional health issues that complicate treatment strategies [14].

### Increased tumor thickness and ulceration

The meta-analysis revealed a statistically significant increase in Breslow thickness during the COVID-19 period (MD = 0.24 mm, 95% CI: 0.02–0.47), which increased to 0.34 mm (95% CI: 0.01–0.66) when focusing on good-quality studies and excluding outliers. While this increase might seem minor, it is clinically significant, as Breslow thickness is a key prognostic indicator. Even slight increases (such as exceeding the 0.8 mm threshold) can influence staging and, consequently, the treatment plan [13]. The high heterogeneity (I² = 92–96%) indicates variations in diagnostic delays, healthcare access, and case-mix across different regions. Additionally, the odds of ulceration were 29% higher (OR = 1.29, 95% CI: 1.15–1.44), rising to 37% in high-quality studies, highlighting a trend towards more advanced diseases. Ulceration, an independent negative prognostic factor, accelerates stage progression and requires more aggressive treatments, like sentinel lymph node biopsy [56]. These findings suggest that diagnostic delays during the pandemic allowed melanomas to progress to thicker, more aggressive forms, potentially compromising survival outcomes.

### Changes in histopathological subtypes

The notable rise in nodular melanoma (OR = 1.34, 95% CI: 1.08–1.67; 1.47 in high-quality studies) is particularly alarming due to its tendency for rapid vertical growth and poor prognosis [57]. The lack of significant changes in superficial spreading, acral lentiginous, or lentigo maligna melanoma indicates that the pandemic disproportionately affected the detection of rapidly progressing, aggressive subtypes. The increase in nodular melanoma cases may be attributed to fewer opportunities for early detection through routine screenings, as these tumors often become symptomatic at more advanced stages. The high heterogeneity (I² = 81–84%) observed in nodular melanoma analyses likely arises from regional variations in healthcare access and referral patterns, which highlights the importance of targeted screening strategies during crises.

### Clinical and public health implications

The combination of fewer diagnoses, thicker tumors, higher ulceration rates, and a rise in nodular melanoma cases suggests a trend toward more advanced melanoma presentations during the COVID-19 period. These developments are likely to lead to increased morbidity, higher treatment expenses, and potentially poorer long-term survival outcomes. While early-stage melanomas have a 5-year survival rate of over 99%, this figure drops to 35% in metastatic cases [58]. The shift in staging could place a significant burden on healthcare systems, as advanced melanomas require more complex surgical interventions, additional therapies, and prolonged follow-up care [12]. Moreover, the high cost of treating advanced-stage melanoma highlights the need to support early detection efforts during healthcare disruptions [59].

Although telemedicine and AI-assisted triage helped address some access barriers during the pandemic, they were less effective at detecting subtle pigmented lesions, particularly in resource-limited countries [22]. Future approaches should combine virtual care with prioritized in-person diagnostic appointments for high-risk individuals, along with public awareness initiatives to promote early assessment of suspicious lesions.

### Strengths and limitations

The strengths of this study include its comprehensive scope (62 studies across multiple continents), rigorous quality assessment using the Newcastle-Ottawa Scale, and sensitivity analyses to ensure robustness. The inclusion of letters and short reports captured valuable data published during the pandemic and enhanced the review’s timeliness. However, high statistical heterogeneity (I² >90% for several outcomes) limits the accuracy of pooled estimates. It reflects the variability in study designs, period definitions, and regional healthcare contexts.

An important limitation of this meta-analysis is the potential for residual confounding. The retrospective nature of the included studies increases the risk of bias from unmeasured factors that may have influenced melanoma diagnosis independently of the pandemic itself, such as comorbidities or insurance status. Although we stratified by country and conducted sensitivity analyses based on study quality, we did not perform meta-regression to explore the effects of study-level covariates, due to incomplete or inconsistent reporting of covariates and substantial heterogeneity across studies.

Only a small subset of included studies reported post-pandemic or recovery-period data, and their definitions of recovery periods varied widely, limiting our ability to systematically analyze post-pandemic diagnostic trends.

### Future directions

To reduce the impact of future healthcare disruptions, policymakers should prioritize investment in resilient dermatology services that enable early detection of aggressive melanomas. Teledermatology, particularly when validated across diverse populations, may improve triage efficiency. Public health initiatives should also promote regular skin self-examinations and encourage timely medical evaluation, especially during periods of crisis. Finally, long-term cohort studies will be essential for evaluating the survival effects of pandemic-related delays and guiding evidence-based resource allocation in melanoma care.

## Conclusion

In conclusion, the COVID-19 pandemic significantly disrupted melanoma diagnosis, leading to fewer diagnoses, thicker and more ulcerated tumors, and a higher prevalence of nodular melanoma. These findings highlight the critical need for robust healthcare strategies to maintain early detection and timely management of melanoma during global health crises, ensuring optimal patient outcomes and system resilience.

## Supplementary Information

Below is the link to the electronic supplementary material.

Supplementary Material 1

Supplementary Material 2

## Data Availability

All data generated or analyzed during this study are included in this published article and its supplementary information files.

## Abbreviations

COVID-19: Coronavirus Disease 2019
IM: Invasive Melanoma
MeSH: Medical Subject Headings
MIS: Melanoma In Situ
NOS: Newcastle-Ottawa Scale
PECO: Population, Exposure, Comparator, Outcome
PRISMA: Preferred Reporting Items for Systematic Reviews and Meta-Analyses
PROSPERO: International Prospective Register of Systematic Reviews
SARS-CoV-2: Severe Acute Respiratory Syndrome Coronavirus 2
WHO: World Health Organization
