# Supplementary material for: The impact of the COVID-19 pandemic on melanoma diagnosis: a systematic review and meta-analysis of global evidence
Source: BMC Public Health. 2025 Aug 6;25:2684. doi: 10.1186/s12889-025-23926-3 (PMC12326724; doi:10.1186/s12889-025-23926-3)
Supplement: Supplementary file 1 — Supplementary Material 1 [file 12889_2025_23926_MOESM1_ESM.docx]

# Search Syntax

Search date: 30 March 2024+ Updated search: 10 September 2024

| **Pubmed** | |
| --- | --- |
| ("Skin Neoplasms" [Mesh Terms] OR (Skin [Title/abstract] AND Neoplasm* [Title/abstract]) OR "Skin Neoplasm" [Title/abstract] OR "Cancer of Skin" [Title/abstract] OR "Skin Cancer*" [Title/abstract] OR "Cancer of the Skin" [Title/abstract] OR (Skin [Title/abstract] AND Cancer* [Title/abstract]) OR "Melanoma" [Mesh] OR "melanomas" [Title/abstract] OR "Malignant Melanoma*" [Title/abstract] OR (Malignant [Title/abstract] AND Melanoma* [Title/abstract])) | #1 |
| ("COVID-19" [Mesh Terms] OR "COVID 19" [Title/abstract] OR "2019-nCoV Infection*" [Title/abstract] OR "2019 nCoV Infection" [Title/abstract] OR (Infection [Title/abstract] AND "2019-nCoV" [Title/abstract]) OR "SARS-CoV-2 Infection*" [Title/abstract] OR (Infection [Title/abstract] AND SARS-CoV-2 [Title/abstract]) OR "SARS CoV 2 Infection" [Title/abstract] OR "2019 Novel Coronavirus Disease" [Title/abstract] OR "2019 Novel Coronavirus Infection" [Title/abstract] OR "COVID-19 Virus Infection*" [Title/abstract] OR "COVID 19 Virus Infection" [Title/abstract] OR (Infection [Title/abstract] AND "COVID-19 Virus" [Title/abstract]) OR ("Virus Infection" [Title/abstract] AND COVID-19 [Title/abstract]) OR COVID19 [Title/abstract] OR "Coronavirus Disease 2019" [Title/abstract] OR ("Disease 2019" [Title/abstract] AND Coronavirus [Title/abstract]) OR "Coronavirus Disease-19" [Title/abstract] OR "Coronavirus Disease 19" [Title/abstract] OR "Severe Acute Respiratory Syndrome Coronavirus 2 Infection" [Title/abstract] OR "COVID-19 Virus Disease*" [Title/abstract] OR "COVID 19 Virus Disease" [Title/abstract] OR (Disease [Title/abstract] AND "COVID-19 Virus" [Title/abstract]) OR ("Virus Disease" [Title/abstract] AND COVID-19 [Title/abstract]) OR "SARS Coronavirus 2 Infection" [Title/abstract] OR "2019-nCoV Disease*" [Title/abstract] OR "2019 nCoV Disease" [Title/abstract] OR (Disease [Title/abstract] AND 2019-nCoV [Title/abstract]) OR "COVID-19 Pandemic*" [Title/abstract] OR "COVID 19 Pandemic" [Title/abstract] OR (Pandemic [Title/abstract] AND COVID-19 [Title/abstract])) | #2 |
| #1 AND #2 | 687 |

| **Scopus** | |
| --- | --- |
| (TITLE-ABS-KEY ("Skin Neoplasms" OR (Skin AND Neoplasm*) OR "Skin Neoplasm" OR "Cancer of Skin" OR "Skin Cancer*" OR "Cancer of the Skin" OR (Skin AND Cancer*) OR "Melanoma" OR "melanomas" OR "Malignant Melanoma*" OR (Malignant AND Melanoma*))) | #1 |
| (TITLE-ABS-KEY ("COVID-19” OR "COVID 19" OR "2019-nCoV Infection*" OR "2019 nCoV Infection" OR (Infection AND "2019-nCoV") OR "SARS-CoV-2 Infection*" OR (Infection AND SARS-CoV-2) OR "SARS CoV 2 Infection" OR "2019 Novel Coronavirus Disease" OR "2019 Novel Coronavirus Infection" OR "COVID-19 Virus Infection*" OR "COVID 19 Virus Infection" OR (Infection AND "COVID-19 Virus") OR ("Virus Infection" AND COVID-19) OR COVID19 OR "Coronavirus Disease 2019" OR ("Disease 2019" AND Coronavirus) OR "Coronavirus Disease-19" OR "Coronavirus Disease 19" OR "Severe Acute Respiratory Syndrome Coronavirus 2 Infection" OR "COVID-19 Virus Disease*" OR "COVID 19 Virus Disease" OR (Disease AND "COVID-19 Virus") OR ("Virus Disease" AND COVID-19) OR "SARS Coronavirus 2 Infection" OR "2019-nCoV Disease*" OR "2019 nCoV Disease" OR (Disease AND 2019-nCoV) OR "COVID-19 Pandemic*" OR "COVID 19 Pandemic" OR (Pandemic AND COVID-19))) | #2 |
| #1 AND #2 | 2392 |

| **Web of Science** | |
| --- | --- |
| (TS=("Skin Neoplasms" OR (Skin AND Neoplasm*) OR "Skin Neoplasm" OR "Cancer of Skin" OR "Skin Cancer*" OR "Cancer of the Skin" OR (Skin AND Cancer*) OR "Melanoma" OR "melanomas" OR "Malignant Melanoma*" OR (Malignant AND Melanoma*))) | #1 |
| (TS=("COVID-19” OR "COVID 19" OR "2019-nCoV Infection*" OR "2019 nCoV Infection" OR (Infection AND "2019-nCoV") OR "SARS-CoV-2 Infection*" OR (Infection AND SARS-CoV-2) OR "SARS CoV 2 Infection" OR "2019 Novel Coronavirus Disease" OR "2019 Novel Coronavirus Infection" OR "COVID-19 Virus Infection*" OR "COVID 19 Virus Infection" OR (Infection AND "COVID-19 Virus") OR ("Virus Infection" AND COVID-19) OR COVID19 OR "Coronavirus Disease 2019" OR ("Disease 2019" AND Coronavirus) OR "Coronavirus Disease-19" OR "Coronavirus Disease 19" OR "Severe Acute Respiratory Syndrome Coronavirus 2 Infection" OR "COVID-19 Virus Disease*" OR "COVID 19 Virus Disease" OR (Disease AND "COVID-19 Virus") OR ("Virus Disease" AND COVID-19) OR "SARS Coronavirus 2 Infection" OR "2019-nCoV Disease*" OR "2019 nCoV Disease" OR (Disease AND 2019-nCoV) OR "COVID-19 Pandemic*" OR "COVID 19 Pandemic" OR (Pandemic AND COVID-19))) | #2 |
| #1 AND #2 | 835 |

| **Embase** | |
| --- | --- |
| ('Skin Neoplasms':ti,ab OR (Skin:ti,ab AND Neoplasm*:ti,ab) OR 'Skin Neoplasm':ti,ab OR 'Cancer of Skin':ti,ab OR 'Skin Cancer*':ti,ab OR 'Cancer of the Skin':ti,ab OR (Skin:ti,ab AND Cancer*:ti,ab) OR 'Melanoma'/exp OR 'melanomas':ti,ab OR 'Malignant Melanoma*':ti,ab OR (Malignant:ti,ab AND Melanoma*:ti,ab)) | #1 |
| (COVID-19 :ti,ab OR 'COVID 19':ti,ab OR '2019-nCoV Infection*':ti,ab OR '2019 nCoV Infection':ti,ab OR (Infection:ti,ab AND '2019-nCoV':ti,ab) OR 'SARS-CoV-2 Infection*':ti,ab OR (Infection:ti,ab AND 'SARS-CoV-2':ti,ab) OR 'SARS CoV 2 Infection':ti,ab OR '2019 Novel Coronavirus Disease':ti,ab OR '2019 Novel Coronavirus Infection':ti,ab OR 'COVID-19 Virus Infection*':ti,ab OR 'COVID 19 Virus Infection':ti,ab OR (Infection:ti,ab AND 'COVID-19 Virus':ti,ab) OR ('Virus Infection':ti,ab AND COVID-19:ti,ab) OR COVID19:ti,ab OR 'Coronavirus Disease 2019':ti,ab OR ('Disease 2019':ti,ab AND Coronavirus:ti,ab) OR 'Coronavirus Disease-19':ti,ab OR 'Coronavirus Disease 19':ti,ab OR 'Severe Acute Respiratory Syndrome Coronavirus 2 Infection':ti,ab OR 'COVID-19 Virus Disease*':ti,ab OR 'COVID 19 Virus Disease':ti,ab OR (Disease:ti,ab AND 'COVID-19 Virus':ti,ab) OR ('Virus Disease':ti,ab AND COVID-19:ti,ab) OR 'SARS Coronavirus 2 Infection':ti,ab OR '2019-nCoV Disease*':ti,ab OR '2019 nCoV Disease':ti,ab OR (Disease:ti,ab AND '2019-nCoV':ti,ab) OR 'COVID-19 Pandemic*':ti,ab OR 'COVID 19 Pandemic':ti,ab OR (Pandemic:ti,ab AND COVID-19:ti,ab)) | #2 |
| #1 AND #2 | 1757 |
