# Supplementary material for: The impact of the COVID-19 pandemic on melanoma diagnosis: a systematic review and meta-analysis of global evidence
Source: BMC Public Health. 2025 Aug 6;25:2684. doi: 10.1186/s12889-025-23926-3 (PMC12326724; doi:10.1186/s12889-025-23926-3)
Supplement: Supplementary file 2 — Supplementary Material 2 [file 12889_2025_23926_MOESM2_ESM.docx]

| **First author, Year** | **Country** | **Era** | **Period Definition** | **Period Duration** | **Newly Diagnosed Melanoma (Male/Female)** | **Newcastle-Ottawa Score** | **Study Quality** |
| --- | --- | --- | --- | --- | --- | --- | --- |
| Aabed, H. 2022 (1) | Romania | Pre-COVID | Jan 1, 2018 - Dec 31, 2019 | 24 | 163 (87/76) | 7 | Good |
|  |  | COVID | Jan 1, 2020 - Jan 31, 2022 | 24 | 138 (70/68) |  |  |
| Apostu, A. P. 2024 (2) | Romania | Pre-COVID | Mar 1, 2019 - Feb 29, 2020 | 12 | 341 (NR) | 7 | Good |
|  |  | COVID | Mar 1, 2020 - Feb 28, 2021  Mar 1, 2021 - Feb 28, 2022  Mar 1, 2022 - Feb 28, 2023 | 36 | 275 (NR) |  |  |
| Asai Y. 2021 (3) | Canada | Pre-COVID | Jan 7, 2019 - Sep 29, 2019 | 8.7 | 910 (NR) | 7 | Good |
|  |  | COVID | Jan 7, 2020 - Sep 29, 2020 | 8.7 | 736 (NR) |  |  |
| Aw, K. 2023 (4) | Canada | Pre-COVID | Mar 1, 2018 - Feb 29, 2020 | 24 | 204 (87/117) | 7 | Good |
|  |  | COVID | Mar 1, 2020 - Mar 22, 2022 | 24.7 | 215 (97/118) |  |  |
| Balakirski, G. 2022 (5) | Germany | Pre-COVID | Jan 1, 2019 - Dec 31, 2019 | 12 | 320 (NR) | 5 | Poor |
|  |  | COVID | Jan 1, 2020 - Dec 31, 2021 | 24 | 319 (NR) |  |  |
| Barcaui, C. B. 2022 (6) | Brazil | Pre-COVID | Jan 1, 2018 - Dec 31, 2019 | 24 | 20 (NR) | 4 | Poor |
|  |  | COVID | Jan 1, 2020 - Dec 31, 2021 | 24 | 16 (NR) |  |  |
| Barruscotti, S. 2020 (7) | Italy | Pre-COVID | Feb 22, 2018 - May 3, 2019 | 14.3 | 42 (NR) | 5 | Poor |
|  |  | COVID | Feb 22, 2020 - May 3, 2020 | 2.5 | 6 (NR) |  |  |
| Bowe, S. 2022 (8) | Ireland | Pre-COVID | Jan 1, 2019 - Dec 31, 2019 | 12 | 52 (NR) | 4 | Poor |
|  |  | COVID | Jan 1, 2020 - Dec 31, 2021 | 24 | 61 (NR) |  |  |
| Cleland, J. B. 2024 (9) | Germany | Pre-COVID | Jun 1, 2018 - Aug 31, 2018 | 3 | 73 (NR) | 6 | Good |
|  |  | COVID | Jun 1, 2022 - Aug 31, 2022 | 3 | 78 (NR) |  |  |
| Cocuz. IG. 2021 (10) | Romania | Pre-COVID | Apr 1, 2019 - Feb 29, 2020 | 11 | 10 (NR) | 7 | Good |
|  |  | COVID | Apr 1, 2020 - Feb 28, 2021 | 11 | 40 (NR) |  |  |
| Davis, C. H. 2022 (11) | USA | Pre-COVID | Aug 1, 2019 - Feb 29, 2020 | 7 | 375 (234/138) | 6 | Fair |
|  |  | COVID | May 1, 2020 - Dec 31, 2020 | 8 | 313 (182/131) |  |  |
| Demaerel, P. G. 2023 (12) | Belgium | Pre-COVID | Jan 1, 2017 - Dec 31, 2019 | 36 | 9647 (NR) | 6 | Good |
|  |  | COVID | Jan 1, 2020 - Dec 31, 2021 | 24 | 3362 (NR) |  |  |
| Drescher, CW. 2021 (13) | USA | Pre-COVID | Mar 4, 2019 – Mar 3, 2020 | 12 | 1185 (NR) | 8 | Good |
|  |  | COVID | Mar 4, 2020 - Mar 3, 2021 | 12 | 953 (NR) |  |  |
| Ferhatosmanoğlu, A. 2024 (14) | Turkey | Pre-COVID | Jan 1, 2018 - Jan 1, 2020 | 24 | 30 (15/15) | 6 | Good |
|  |  | COVID | Jun 1, 2022 - Jan 1, 2023 | 7 | 37 (19/18) |  |  |
| Ferrara, G. 2020 (15) | Italy | Pre-COVID | Mar 12, 2018 - May 20, 2019 | 4.6 | 181 (NR) | 6 | Fair |
|  |  | COVID | Mar 12, 2020 - May 20, 2020 | 2.3 | 92 (NR) |  |  |
| Filoni, A. 2021 (16) | Italy | Pre-COVID | Feb 23, 2019 - May 21, 2019 | 2.9 | 66 (NR) | 5 | Poor |
|  |  | COVID | Feb 23, 2020 - May 21, 2020 | 2.9 | 64 (NR) |  |  |
| Gaunt, N. 2021 (17) | UK | Pre-COVID | Oct 1, 2019 - Dec 31, 2019 | 3 | 71 (NR) | 5 | Poor |
|  |  | COVID | Oct 1, 2020 - Dec 31, 2020 | 3 | 52 (NR) |  |  |
| Gedeah, C. 2021 (18) | Belgium | Pre-COVID | Mar 15, 2018 - Dec 31, 2019 | 21.5 | 471 (NR) | 4 | Poor |
|  |  | COVID | Mar 15, 2020 - Dec 31, 2020 | 9.5 | 161 (NR) |  |  |
| Gil-Pallares, P 2023 1^st^ (19) | Spain | Pre-COVID | Mar 14, 2019 - Sep 13, 2019 | 6 | 29 (14/15) | 6 | Fair |
|  |  | COVID | Sep 14, 2019 - Mar 13, 2020 | 6 | 24 (11/13) |  |  |
| Gil-Pallares, P 2023 2^nd^ (19) | Spain | Pre-COVID | Mar 14, 2020 - Sep 13, 2020 | 6 | 36 (11/25) |  |  |
|  |  | COVID | Sep 14, 2021 - Mar 13, 2021 | 6 | 30 (11/19) |  |  |
| Gisondi, P. 2021 (20) | Italy | Pre-COVID | Mar 1, 2019 - Oct 31, 2019 | 8 | 634 (351/283) | 7 | Poor |
|  |  | COVID | Mar 1, 2020 - Oct 31, 2020 | 8 | 556 (314/242) |  |  |
| Granahan, A. 2022 (21) | France | Pre-COVID | Mar 1, 2018 - Aug 31, 2018 | 6 | 243 (NR) | 4 | Fair |
|  |  | COVID | Mar 1, 2020 - Aug 31, 2020 | 6 | 217 (NR) |  |  |
| Gualdi, G. 2021 (22) | Italy | Pre-COVID | May 1, 2017 - Jul 31, 2019 | 9 | 296 (147/149) | 8 | Good |
|  |  | COVID | May 1, 2020 - Jul 31, 2020 | 3 | 237 (115/122) |  |  |
| Guven, D. C. 2021 (23) | Turkey | Pre-COVID | Mar 1, 2019 - Dec 31, 2019 | 10 | 27 (NR) | 6 | Poor |
|  |  | COVID | Mar 1, 2020 - Dec 31, 2020 | 10 | 15 (NR) |  |  |
| Hanuschak, D. 2024 (24) | USA | Pre-COVID | May 1, 2018 - Feb 29, 2020 | 22 | 69 (NR) | 5 | Poor |
|  |  | COVID | Apr 1, 2020 - Jan 31, 2022 | 22 | 32 (NR) |  |  |
| Heath, H. 2022 (25) | UK | Pre-COVID | Nov 1, 2018 - Feb 29, 2020 | 16 | 276 (135/141) | 5 | Poor |
|  |  | COVID | Mar 1, 2020 - Jun 30, 2021 | 16 | 242 (118/124) |  |  |
| Hoellwerth, M. 2021 (26) | Austria | Pre-COVID | Mar 16, 2018 - Jun 10, 2019 | 5.7 | 505 (240/260) | 6 | Good |
|  |  | COVID | Mar 16, 2020 - Jun 10, 2020 | 2.8 | 432 (199/233) |  |  |
| Hurley, C. M. 2022 (27) | Ireland | Pre-COVID | Mar 1, 2019 - Dec 31, 2019 | 10 | 277 (137/140) | 6 | Poor |
|  |  | COVID | Mar 1, 2020 - Dec 31, 2020 | 10 | 312 (146/166) |  |  |
| Ibrahim, L. S. 2023 (28) | UK | Pre-COVID | Jan 1, 2019 - Dec 31, 2019 | 12 | 14001 (NR) | 7 | Poor |
|  |  | COVID | Jan 1, 2020 - Dec 31, 2021 | 24 | 11516 (NR) |  |  |
| Javor, S. 2021 (29) | Italy | Pre-COVID | Jan 1, 2019 - Dec 31, 2019 | 12 | 138 (NR) | 5 | Poor |
|  |  | COVID | Jan 1, 2020 - Dec 31, 2020 | 12 | 87 (NR) |  |  |
| Jeremic, J. 2022 (30) | Serbia | Pre-COVID | Jan 1, 2017 – Mar 14, 2020 | 38.5 | 339 (189/150) | 7 | Good |
|  |  | COVID | Mar 15, 2020 - Mar 31, 2022 | 24.5 | 54 (30/24) |  |  |
| Kinslow, C. J. 2024 (31) | USA | Pre-COVID | Jan 1, 2019 - Dec 31, 2019 | 12 | 63087 (NR) | 8 | Good |
|  |  | COVID | Jan 1, 2020 - Dec 31, 2020 | 12 | 49802 (NR) |  |  |
| Kleemann, J. 2022 (32) | Germany | Pre-COVID | Mar 18, 2019 – Mar 17, 2020 | 12 | 35037 (19301/16730) | 7 | Good |
|  |  | COVID | Mar 18 2020 – Mar 17, 2021 | 12 | 32189 (17401/14786) |  |  |

| Klepfisch, L., 2023 (33) | France | Pre-COVID | Aug 29, 2019 – Mar 16, 2020 | 6.5 | 490 (267/223) | 6 | Poor |
| --- | --- | --- | --- | --- | --- | --- | --- |
|  |  | COVID | Mar 17, 2020 - Nov 28, 2020 | 8.4 | 556 (307/249) |  |  |
| Koch, E., 2021 (34) | Chile | Pre-COVID | Jan 1, 2019 – Mar 31, 2020 | 15 | 191 (80/111) | 5 | Poor |
|  |  | COVID | Apr 1, 2020 - Mar 31, 2021 | 12 | 105 (54/51) |  |  |
| Kostner, L., 2022 (35) | Switzerland | Pre-COVID | Feb 1, 2019 – Mar 15, 2020 | 13.5 | 654 (383/271) | 8 | Good |
|  |  | COVID | Mar 16, 2020 - Apr 30, 2021 | 13.5 | 580 (354/226) |  |  |
| Lamm, R., 2022 (36) | USA | Pre-COVID | May 1, 2019 – May 31, 2020 | 12 | 51 (32/19) | 7 | Good |
|  |  | COVID | Jun 1, 2020 – Sep 30, 2021 | 16 | 61 (32/29) |  |  |
| Makaranka, S., 2022 (37) | Scotland and North Cancer Alliance (NCA) | Pre-COVID | Jan 1, 2019 - Dec 31, 2019 | 12 | 1950 (NR) | 8 | Good |
|  |  | COVID | Jan 1, 2020 - Dec 31, 2020 | 12 | 1605 (NR) |  |  |
| Martinez-Lopez, A., 2022 (38) | Spain | Pre-COVID | Mar 15, 2019 – Mar 14, 2020 | 12 | 77 (43/34) | 7 | Good |
|  |  | COVID | Mar 15, 2020 - Mar 14, 2021 | 12 | 53 (23/30) |  |  |
| Marusca, G., 2024 (39) | USA | Pre-COVID | Sep 15, 2019 - Mar 14, 2020 | 6 | 347 (NR) | 8 | Good |
|  |  | COVID | Mar 15, 2020 – Dec 14, 2020 | 9 | 455 (NR) |  |  |
| McClean, A., 2022 (40) | UK | Pre-COVID | Apr 1, 2019 - Oct 31, 2019 | 7 | 109 (49/60) | 6 | Good |
|  |  | COVID | Apr 1, 2020 - Oct 31, 2020 | 7 | 74 (32/42) |  |  |
| Morais, S., 2021 (41) | Portugal | Pre-COVID | Mar 2, 2019 - Jul 1, 2019 | 4 | 80 (NR) | 6 | Fair |
|  |  | COVID | Mar 2, 2020 - Jul 1, 2020 | 4 | 56 (NR) |  |  |
| Nikolić, J., 2024 (42) | Serbia | Pre-COVID | Jan 1, 2017 - Dec 31, 2019 | 36 | 356 (179/177) | 6 | Fair |
|  |  | COVID | Jan 1, 2020 - Dec 31, 2022 | 36 | 273 (139/134) |  |  |
| Pagliarello, C., 2023 (43) | Italy | Pre-COVID | Mar 9, 2019 – Mar 9, 2020 | 12 | 402 (212/190) | 8 | Good |
|  |  | COVID | Mar 9, 2020 - Mar 9, 2021 | 12 | 245 (117/128) |  |  |
| Pissa, M., 2022 (44) | Sweden | Pre-COVID | Apr 1, 2019 – Mar 31, 2020 | 12 | 126 (NR) | 6 | Poor |
|  |  | COVID | Apr 1, 2020 - Mar 31, 2021 | 12 | 118 (NR) |  |  |
| Sangers, T. E., 2022 (45) | Netherlands | Pre-COVID | Jan 1, 2019 – Mar 11, 2020 | 14 | 9377 (4704/4673) | 7 | Good |
|  |  | COVID | Mar 12, 2020 - Jul 22, 2021 | 16.4 | 11057 (NR) |  |  |
| Sanvisens, A., 2021 (46) | Spain | Pre-COVID | Jan 1, 2019 - Feb 29, 2020 | 14 | 44 (NR) | 7 | Good |
|  |  | COVID | Mar 1, 2020 - Dec 31, 2020 | 10 | 39 (NR) |  |  |
| Sarriugarte Aldecoa, C., 2022 (47) | Spain | Pre-COVID | Mar 1, 2018 – Oct 31, 2018 & Mar 1, 2019 – Oct 31, 2019 | 16 | 155 (NR) | 6 | Poor |
|  |  | COVID | Mar 1, 2020 - Oct 31, 2020 | 8 | 55 (NR) |  |  |
| Sazali, H. B. 2021 (48) | Ireland | Pre-COVID | Jan 1, 2019 - Dec 31, 2019 | 12 | 105 (NR) | 6 | Poor |
|  |  | COVID | Jan 1, 2020 - Dec 31, 2020 | 12 | 65 (NR) |  |  |
| Scharf, C. 2022 (49) | Europe | Pre-COVID | Mar 1, 2019 - Feb 29, 2020 | 12 | 2311 (NR) | 7 | Poor |
|  |  | COVID | Mar 1, 2020 - Feb 28, 2021 | 12 | 1722 (NR) |  |  |
| Schauer, A. A. 2020 (50) | UK | Pre-COVID | Jan 27, 2020 – Mar 22, 2020 | 1.8 | 8 (NR) | 6 | Poor |
|  |  | COVID | Mar 23, 2020 - May 18, 2020 | 1.9 | 9 (NR) |  |  |
| Seretis, K. 2021 (51) | Greece | Pre-COVID | May 20, 2019 - Sep 20, 2019 | 4 | 22 (NR) | 6 | Good |
|  |  | COVID | May 20, 2020 - Sep 20, 2020 | 4 | 25 (NR) |  |  |
| Shaikh, S. S. 2023 (52) | USA | Pre-COVID | Mar 1, 2019 – Mar 10, 2020 | 12.3 | 246 (130/116) | 6 | Poor |
|  |  | COVID | Mar 11, 2020 – Jan 12, 2021 | 10 | 246 (137/109) |  |  |
| Skowron, F. 2023 (53) | France | Pre-COVID | Mar 17, 2019 – Mar 16, 2020 | 12 | 1119 (597/522) | 6 | Poor |
|  |  | COVID | May 11, 2020 - May 10, 2021 | 12 | 1018 (506/512) |  |  |
| Spurny-Dworak, J. 2022 (54) | Austria | Pre-COVID | Mar 1, 2019 - Feb 29, 2020 | 12 | 227 (121/106) | 5 | Poor |
|  |  | COVID | Mar 1, 2020 - Feb 28, 2021 | 12 | 201 (132/69) |  |  |
| Topyildiz, H. 2023 (55) | Turkey | Pre-COVID | Dec 20, 2019 – Dec 13, 2020 | 2 | 1 (NR) | 6 | Good |
|  |  | COVID | Mar 16, 2020 - Jun 16, 2020 | 2 | 8 (NR) |  |  |
| Trepanowski, N. 2022 (56) | USA | Pre-COVID | Mar 1, 2019 - Feb 29, 2020 | 12 | 2062 (NR) | 9 | Good |
|  |  | COVID | Mar 1, 2020 - Feb 28, 2021 | 12 | 1834 (NR) |  |  |
| Troesch, A. 2023 (57) | Switzerland, Germany, Austria, Italy | Pre-COVID | Sep 1, 2018 - Feb 29, 2020 | 18 | 4340 (2302/2038) | 7 | Good |
|  |  | COVID | Mar 1, 2020 - Aug 31, 2021 | 18 | 3525 (1866/1659) |  |  |
| Ungureanu, L. 2022 (58) | Romania | Pre-COVID | Mar 1, 2019 - Feb 29, 2020 | 12 | 341 (164/177) | 7 | Good |
|  |  | COVID | Mar 1, 2020 - Feb 28, 2021 | 12 | 275 (138/137) |  |  |
| Voigtländer, S. 2023 (59) | Germany | Pre-COVID | Mar 1, 2019 - Feb 29, 2020 | 12 | 1392 (NR) | 7 | Good |
|  |  | COVID | Mar 1, 2020 - Feb 28, 2021 | 12 | 1158 (NR) |  |  |
| Wang, R. 2020 (60) | Germany | Pre-COVID | Jan 6, 2015 - Apr 15, 2019 | 25 | 17 (NR) | 6 | Good |
|  |  | COVID | Jan 7, 2020 - Apr 13, 2020 | 3.2 | 3 (NR) |  |  |
| Welzel, J. 2022 (61) | Germany | Pre-COVID | Jan 1, 2019 – Jan 31, 2019 &  Jan 1, 2020 – Jan 31, 2020 | 2 | 646 (NR) | 5 | Poor |
|  |  | COVID | Jan 1, 2021 - Jan 31, 2021 | 1 | 56 (NR) |  |  |
| Śmigielska, P. 2023 (62) | Poland | Pre-COVID | Mar 1, 2018 - Jun 30, 2019 | 16 | 66 (27/39) | 5 | Poor |
|  |  | COVID | Mar 1, 2020 - Jun 30, 2021 | 16 | 52 (14/38) |  |  |

**Supplementary Table S2.** Characteristics of included studies comparing pre-COVID and COVID-era melanoma diagnoses with male/female breakdown where available, period definitions, and duration (in months) NOS = Newcastle–Ottawa Scale. Quality ratings were converted based on AHRQ thresholds.

**References:**

1. Aabed H, Bloanca V, Crainiceanu Z, Bratosin F, Citu C, Diaconu MM, et al. The Impact of SARS-CoV-2 Pandemic on Patients with Malignant Melanoma at a Romanian Academic Center: A Four-Year Retrospective Analysis. International Journal of Environmental Research and Public Health. 2022;19(14).

2. Apostu AP, Vesa Ș C, Frățilă S, Iancu G, Bejinariu N, Muntean M, et al. The effects of the COVID-19 pandemic on the diagnosis and prognosis of melanoma 2 years after the pandemic in two Romanian counties. Front Med (Lausanne). 2024;11:1328488.

3. Asai Y, Nguyen P, Hanna TP. Impact of the COVID-19 pandemic on skin cancer diagnosis: A population-based study. PLoS ONE. 2021;16(3 March 2021).

4. Aw K, Lau R, Nessim C. Prioritizing Melanoma Surgeries to Prevent Wait Time Delays and Upstaging of Melanoma during the COVID-19 Pandemic. Current Oncology. 2023;30(9):8328-37.

5. Balakirski G, Michalowitz AL, Kreuter A, Hofmann SC. Long-term effects of the COVID-19 pandemic on malignant melanoma: increased lymph node metastases in two German dermatology clinics. Journal of the European Academy of Dermatology and Venereology. 2022;36(10):e762-e4.

6. Barcaui CB, Machado CJ, Piñeiro-Maceira J. Impact of the SARS-CoV-2 pandemic on the diagnosis of primary cutaneous melanoma at a University Hospital in Rio de Janeiro. Anais Brasileiros de Dermatologia. 2022;97(6):801-3.

7. Barruscotti S, Giorgini C, Brazzelli V, Vassallo C, Michelerio A, Klersy C, et al. A significant reduction in the diagnosis of melanoma during the COVID-19 lockdown in a third-level center in the Northern Italy. Dermatologic Therapy. 2020;33(6).

8. Bowe S, Wolinska A, Murray G, Malone C, Feighery C, Roche M. The influence of the COVID-19 pandemic on Breslow thickness of tumours and provision of outpatient malignant melanoma services in an Irish dermatology centre. Clinical and Experimental Dermatology. 2022;47(6):1193-4.

9. Cleland JB, Greenzaid JD, Doerfler L, Ahn CS. Wait times for surgery of cutaneous malignancies following the COVID-19 pandemic: a retrospective cohort analysis. Archives of Dermatological Research. 2024;316(7).

10. Cocuz IG, Cocuz ME, Niculescu R, Șincu MC, Tinca AC, Sabău AH, et al. The Impact of and Adaptations Due to the COVID-19 Pandemic on the Histopathological Diagnosis of Skin Pathologies, Including Non-Melanocyte and Melanoma Skin Cancers-A Single-Center Study in Romania. Medicina (Kaunas). 2021;57(6).

11. Davis CH, Ho J, Greco SH, Koshenkov VP, Vidri RJ, Farma JM, Berger AC. COVID-19 is Affecting the Presentation and Treatment of Melanoma Patients in the Northeastern United States. Annals of Surgical Oncology. 2022;29(3):1629-35.

12. Demaerel PG, Leloup A, Brochez L, Van Eycken L, Garmyn M. Impact of the COVID-19 Pandemic on the Incidence and Thickness of Cutaneous Melanoma in Belgium. Biomedicines. 2023;11(6).

13. Drescher CW, Bograd AJ, Chang SC, Weerasinghe RK, Vita A, Bell RB. Cancer case trends following the onset of the COVID-19 pandemic: A community-based observational study with extended follow-up. Cancer. 2022;128(7):1475-82.

14. Ferhatosmanoğlu A, Selcuk LB, Ersöz Ş, Çelik EK, Keskin F, Arıca DA. Changes in the Clinical and Histopathological Features of Melanoma and Nonmelanoma Skin Cancers after COVID-19 Lockdown Period. Turkish Journal of Dermatology. 2023;17(4):119-25.

15. Ferrara G, De Vincentiis L, Ambrosini-Spaltro A, Barbareschi M, Bertolini V, Contato E, et al. Cancer diagnostic delay in northern and central Italy during the 2020 lockdown due to the coronavirus disease 2019 pandemic: assessment of the magnitude of the problem and proposals for corrective actions. American journal of clinical pathology. 2021;155(1):64-8.

16. Filoni A, Del Fiore P, Cappellesso R, Dall'Olmo L, Salimian N, Spina R, et al. Management of melanoma patients during COVID-19 pandemic in an Italian skin unit. Dermatol Ther. 2021;34(3):e14908.

17. Gaunt N, Green RL, Motta LF, Jamieson LA. Skin cancers in lockdown: no impact on pathological tumour staging. British Journal of Dermatology. 2021;185(4):844-6.

18. Gedeah C, Damsin T, Absil G, Somja J, Collins P, Rorive A, et al. The impact of COVID-19 on the new diagnoses of melanoma. European Journal of Dermatology. 2021;31(4):565-7.

19. Gil-Pallares P, Figueroa-Silva O, Gil-Pallares ME, Vázquez-Bueno J, Piñeyro-Molina F, Monteagudo B, Heras-Sotos CL. Did COVID-19 lockdown delay actually worsen melanoma prognosis? An Bras Dermatol. 2023;98(2):176-80.

20. Gisondi P, Cazzaniga S, Di Leo S, Piaserico S, Bellinato F, Pizzolato M, et al. Impact of the COVID-19 pandemic on melanoma diagnosis. Journal of the European Academy of Dermatology and Venereology. 2021;35(11):e714-e5.

21. Granahan A, Sazali H, Tummon O, Costigan O, Fleming L, Moriarty B, Lally A. The ‘number needed to treat’ metric: a further marker of the impact of COVID-19 on malignant melanomas. Clinical and Experimental Dermatology. 2022;47(7):1377-9.

22. Gualdi G, Porreca A, Amoruso GF, Atzori L, Calzavara-Pinton P, De Tursi M, et al. The Effect of the COVID-19 Lockdown on Melanoma Diagnosis in Italy. Clin Dermatol. 2021;39(5):911-9.

23. Guven DC, Sahin TK, Yildirim HC, Cesmeci E, Incesu FGG, Tahillioglu Y, et al. Newly diagnosed cancer and the COVID-19 pandemic: tumour stage migration and higher early mortality. BMJ supportive & palliative care. 2021.

24. Hanuschak D, DePiero M, DeMoraes M, Bailly S, Rubens M, Lindeman P, et al. The impact of COVID-19 on patients diagnosed with melanoma, breast, and colorectal cancer. American Journal of Surgery. 2023.

25. Heath HT, McGrath EJ, Acheson P. The effect of lockdown on melanoma stage in Devon, UK. Clin Exp Dermatol. 2022;47(8):1581-2.

26. Hoellwerth M, Kaiser A, Emberger M, Brandlmaier M, Laimer M, Egger A, et al. Covid‐19‐induced reduction in primary melanoma diagnoses: Experience from a dermatopathology referral center. Journal of Clinical Medicine. 2021;10(18).

27. Hurley CM, Wrafter L, Dhannoon A, Regan H, Regan PJ. Optimising the Management of Malignant Melanoma during COVID-19. JPRAS Open. 2022;31:72-5.

28. Ibrahim LS, Venables ZC, Levell NJ. The impact of COVID-19 on dermatology outpatient services in England in 2020. Clinical and Experimental Dermatology. 2021;46(2):377-8.

29. Javor S, Sola S, Chiodi S, Brunasso AMG, Massone C. COVID-19-related consequences on melanoma diagnoses from a local Italian registry in Genoa, Italy. International Journal of Dermatology. 2021;60(9):e336-e7.

30. Jeremić J, Suđecki B, Radenović K, Mihaljević J, Radosavljević I, Jovanović M, et al. Impact of the COVID-19 Pandemic on Melanoma Diagnosis: Increased Breslow Thickness in Primary Melanomas—A Single Center Experience. International Journal of Environmental Research and Public Health. 2022;19(24).

31. Kinslow CJ, DeStephano DM, Neugut AI, Taparra K, Horowitz DP, Yu JB, Cheng SK. Site-specific patterns of early-stage cancer diagnosis during the COVID-19 pandemic. JNCI Cancer Spectrum. 2024;8(3):pkae022.

32. Kleemann J, Meissner M, Özistanbullu D, Balaban Ü, Old O, Kippenberger S, et al. Impact of the Covid-19 pandemic on melanoma and non-melanoma skin cancer inpatient treatment in Germany – a nationwide analysis. Journal of the European Academy of Dermatology and Venereology. 2022;36(10):1766-73.

33. Klepfisch L, Carbonnelle-Puscian A, Faisant M, Godeneche J, Provencal N, Lacoste C, Skowron F. Impact of the COVID-19 lockdown on the severity of newly-diagnosed primary cutaneous melanoma: A retrospective regional study in France. Annales de Dermatologie et de Venereologie. 2023;150(1):49-51.

34. Koch E, Villanueva F, Marchetti MA, Abarzúa-Araya Á, Cárdenas C, Castro JC, et al. Reduction in the number of early melanomas diagnosed during the COVID-19 pandemic: a single-centre cohort study. Journal of the European Academy of Dermatology and Venereology. 2021;35(11):e735-e7.

35. Kostner L, Cerminara SE, Pamplona GSP, Maul JT, Dummer R, Ramelyte E, et al. Effects of COVID-19 Lockdown on Melanoma Diagnosis in Switzerland: Increased Tumor Thickness in Elderly Females and Shift towards Stage IV Melanoma during Lockdown. Cancers. 2022;14(10).

36. Lamm R, Lyons W, So W, Willis AI. Advanced-Stage Melanoma at Presentation Following the Peak of the Pandemic: A COVID-19 Cancer Canary in a Coal Mine. World Journal of Surgery. 2022;46(8):1820-5.

37. Makaranka S, Scutt F, Rahman K. The Impact of the COVID-19 Pandemic on Diagnosis of Skin Cancer Cases in North Cancer Alliance and Scotland. Cureus Journal of Medical Science. 2022;14(5).

38. Martinez-Lopez A, Diaz-Calvillo P, Cuenca-Barrales C, Montero-Vilchez T, Sanchez-Diaz M, Buendia-Eisman A, Arias-Santiago S. Impact of the COVID-19 Pandemic on the Diagnosis and Prognosis of Melanoma. Journal of Clinical Medicine. 2022;11(14).

39. Marusca G, Dlugas H, Kim S, Ruterbusch JJ, Duque D, Neill D, et al. Median Breslow thickness during, post and pre-COVID-19 lockdown and correlation with socioeconomic factors. Journal of Clinical Oncology. 2024;42(16).

40. McClean A, Matteucci P, Totty J. The impact of COVID19 on the presentation, diagnosis and management of cutaneous melanoma and squamous cell carcinoma in a single tertiary referral centre. Journal of Plastic, Reconstructive and Aesthetic Surgery. 2022;75(8):2831-70.

41. Morais S, Antunes L, Rodrigues J, Fontes F, Bento MJ, Lunet N. The impact of the COVID-19 pandemic on the short-term survival of patients with cancer in Northern Portugal. International Journal of Cancer. 2021;149(2):287-96.

42. Nikolić J, Marinković M, Jovanović M, Mijatov I, Leković-Stojanov D, Kecman S. Insight into the management of patients with melanoma in times of the COVID-19 pandemic – a single-center experience. Vojnosanitetski Pregled. 2024;81(4):197-202.

43. Pagliarello C, Sicher M, Girardelli CR, Stanganelli I. Prolonged impact of COVID-19 pandemic on delayed melanoma diagnosis: Further data based on one-year appraisal from Italy. Dermatology Reports. 2023;15(1).

44. Pissa M, Gulin SJ. The incidence of melanoma during the COVID-19 pandemic in a Swedish health care region without lockdown. JAAD Int. 2022;6:82-3.

45. Sangers TE, Wakkee M, Kramer-Noels EC, Nijsten T, Louwman MWJ, Jaspars EH, Hollestein LM. Limited impact of COVID-19-related diagnostic delay on cutaneous melanoma and squamous cell carcinoma tumour characteristics: a nationwide pathology registry analysis. British Journal of Dermatology. 2022;187(2):196-202.

46. Sanvisens A, Puigdemont M, Rubió-Casadevall J, Vidal-Vila A, López-Bonet E, Martín-Romero F, Marcos-Gragera R. Differences in the impact of covid-19 on pathology laboratories and cancer diagnosis in girona. International Journal of Environmental Research and Public Health. 2021;18(24).

47. Sarriugarte Aldecoa-Otalora J, Loidi Pascual L, Córdoba Iturriagagoitia A, Yanguas Bayona JI. [Translated article] How Has the COVID-19 Pandemic and Lockdown Affected Breslow Thickness in Cutaneous Melanoma? Actas Dermo-Sifiliograficas. 2022;113(1):T107-T9.

48. Sazali HB, Roche L, Alsharqi A, Kirby B, Moriarty B, Lally A. Decline in pigmented lesion referrals and melanoma diagnoses during covid-19 lockdown. Irish Medical Journal. 2021;114(6).

49. Scharf C, Brancaccio G, Di Stefani A, Fargnoli MC, Kittler H, Kyrgidis A, et al. The association between COVID-19 lockdowns and melanoma diagnosis and thickness: A multicenter retrospective study from Europe. Journal of the American Academy of Dermatology. 2022;87(3):648-9.

50. Schauer AA, Kulakov EL, Martyn-Simmons CL, Bunker CB, Edmonds EVJ. Melanoma defies ‘lockdown’: ongoing detection during Covid-19 in central London. Clinical and Experimental Dermatology. 2020;45(7):900.

51. Seretis K, Boptsi E, Boptsi A, Lykoudis EG. The impact of treatment delay on skin cancer in COVID-19 era: a case-control study. World Journal of Surgical Oncology. 2021;19(1).

52. Shaikh SS, Yang X, Fortman DD, Wang H, Davar D, Luke JJ, et al. A retrospective analysis of the impact of the COVID-19 pandemic on staging at presentation of patients with invasive melanoma. Journal of the American Academy of Dermatology. 2022.

53. Skowron F, Mouret S, Seigneurin A, Montaudié H, Maubec E, Grange F, et al. Impact of the Covid-19 pandemic on melanoma diagnosis: A retrospective study from the French clinical database of melanoma patients (RIC-Mel). JEADV Clinical Practice. 2023;2(3):638-42.

54. Spurny-Dworak J, Steiner A, Breier F, Stella A, Feldmann R. Effects of the COVID-19 pandemic on the diagnosis of malignant melanoma: a retrospective study. European Journal of Dermatology. 2022;32(5):615-7.

55. Topyıldız H, Şavk E, Uslu M, Güven M. The effects of the COVID-19 pandemic on the Dermatology Outpatient Clinic of Aydın Adnan Menderes University Hospital. Turkderm Turkish Archives of Dermatology and Venereology. 2023;57(3):119-26.

56. Trepanowski N, Chang MS, Zhou G, Ahmad M, Berry EG, Bui K, et al. Delays in melanoma presentation during the COVID-19 pandemic: A nationwide multi-institutional cohort study. Journal of the American Academy of Dermatology. 2022;87(5):1217-9.

57. Troesch A, Hoellwerth M, Forchhammer S, Del Regno L, Lodde G, Turko P, et al. The impact of the COVID-19 pandemic on the diagnosis of cutaneous melanomas: A retrospective cohort study from five European skin cancer reference centres. Journal of the European Academy of Dermatology and Venereology. 2023;37(5):922-31.

58. Ungureanu L, Apostu AP, Vesa Ș C, Cășeriu AE, Frățilă S, Iancu G, et al. Impact of the COVID-19 Pandemic on Melanoma Diagnosis in Romania-Data from Two University Centers. Int J Environ Res Public Health. 2022;19(22).

59. Voigtländer S, Hakimhashemi A, Grundmann N, Radespiel-Tröger M, Inwald EC, Ortmann O, et al. Impact of the COVID-19 pandemic on reported cancer diagnoses in Bavaria, Germany. Journal of Cancer Research and Clinical Oncology. 2023;149(10):7493-503.

60. Wang R, Helf C, Tizek L, Neuhauser R, Eyerich K, Zink A, et al. The impact and consequences of sars-cov-2 pandemic on a single university dermatology outpatient clinic in Germany. International Journal of Environmental Research and Public Health. 2020;17(17):1-10.

61. Welzel J, Augustin M, Gutzmer R. Impact of the COVID-19 pandemic on the care of patients with malignant melanoma. JDDG - Journal of the German Society of Dermatology. 2022;20(7):1028-30.

62. Śmigielska P, Sławińska M, Sikorska M, Sobjanek M. The impact of the COVID-19 pandemic on the characteristics of melanoma: a single-centre cohort study. Postepy Dermatologii i Alergologii. 2023;40(5):638-41.
